# METTL3-mediated m^6^A mRNA modification of FBXW7 suppresses lung adenocarcinoma

**DOI:** 10.1186/s13046-021-01880-3

**Published:** 2021-03-06

**Authors:** Yingtong Wu, Ning Chang, Yong Zhang, Xinxin Zhang, Leidi Xu, Yinggang Che, Tianyun Qiao, Bin Wu, Ying Zhou, Jun Jiang, Jie Xiong, Jian Zhang, Jian Zhang

**Affiliations:** 1 Department of Respiratory Diseases, Xijing Hospital, Fourth Military Medical University, Chang-Le Xi Street #127, Xi’an, 710032 People’s Republic of China; 2 State Key Laboratory of Cancer Biology, Department of Biochemistry and Molecular Biology, Fourth Military Medical University, Chang-Le Xi Street #169, Xi’an, 710032 People’s Republic of China; 3 Department of Thoracic Surgery, Tangdu Hospital, Fourth Military Medical University, 1 Xinsi Road, Baqiao District, Xi’an, Shaanxi 710038 People’s Republic of China

**Keywords:** METTL3, FBXW7, Lung adenocarcinoma, m^6^A, Epigenetic modification

## Abstract

**Background:**

FBXW7 m^6^A modification plays an important role in lung adenocarcinoma (LUAD) progression; however, the underlying mechanisms remain unclear.

**Methods:**

The correlation between FBXW7 and various genes related to m^6^A modification was analyzed using The Cancer Genome Atlas database. The regulatory effects of METTL3 on FBXW7 mRNA m^6^A modification were examined in a cell model, and the underlying mechanism was determined by methylated RNA immunoprecipitation, RNA immunoprecipitation, luciferase reporter, and mutagenesis assays. In vitro experiments were performed to further explore the biological effects of METTL3-mediated FBXW7 m^6^A modification on LUAD development.

**Results:**

Decreased FBXW7 expression was accompanied by downregulated METTL3 expression in human LUAD tissues and was associated with a worse prognosis for LUAD in The Cancer Genome Atlas database. m^6^A was highly enriched in METTL3-mediated FBXW7 transcripts, and increased m^6^A modification in the coding sequence region increased its translation. Functionally, METTL3 overexpression or knockdown affected the apoptosis and proliferation phenotype of LUAD cells by regulating FBXW7 m^6^A modification and expression. Furthermore, FBXW7 overexpression in METTL3-depleted cells partially restored LUAD cell suppression in vitro and in vivo.

**Conclusions:**

Our findings reveal that METTL3 positively regulates FBXW7 expression and confirm the tumor-suppressive role of m^6^A-modified FBXW7, thus providing insight into its epigenetic regulatory mechanisms in LUAD initiation and development.

**Supplementary Information:**

The online version contains supplementary material available at 10.1186/s13046-021-01880-3.

## Background

With approximately 2 million new cases and 1.5 million related deaths recorded per year, lung cancer accounts for the highest percentage of cancer morbidity and mortality worldwide [1]. In recent decades, lung adenocarcinoma (LUAD) has become the primary histological subtype of non-small cell lung cancer (NSCLC), which is the major pathological type of lung cancer [2]. Despite recent improvements in diagnosis and treatment strategies, the survival rate of patients with LUAD remains low; therefore, it is vital to understand the molecular mechanisms underlying tumorigenesis to prevent the development of LUAD during the early stages.

F-box and WD repeat domain-containing 7 (FBXW7), also known as Fbw7, Sel10, hCDC4, or hAgo, is a well-established substrate recognition subunit of the SCF (SKP1–CUL1–F-box protein) E3 ubiquitin ligase complex [3]. High FBXW7 expression is independently associated with a favorable prognosis in patients with NSCLC, as it plays a negative role in NSCLC pathogenesis [4]. Accumulating studies have shown that FBXW7 dysregulation by point mutation, genomic deletion, or promoter hypermethylation leads to various cancers [5] and that Fbxw7^+/−^ mice are more prone to radiation-induced tumorigenesis [6, 7]. FBXW7 is regulated by various pathways including tumor suppressor p53 [8], Pin1 (peptidyl-prolyl cis-trans isomerase NIMA-interacting1) [9], C/EBP-δ (CCAAT/enhancer-binding protein-δ), Hes-5 (hairy and enhancer-of-split homologs 5) [10], and microRNAs [11]. However, the m^6^A modifications governing FBXW7 stability and/or function in cancers and how FBXW7 expression may be modulated by m^6^A modification in lung cancer remain largely unknown.

Identification of cancer-related epigenetic modifications is a rapidly expanding field of study within molecular biological mechanisms, and research has indicated that RNA-targeted modifications are biologically crucial in tumorigenesis [12]. Among the more than 170 known RNA modifications, N6-methyladenosine (m^6^A) is the most abundant internal mRNA modification and reportedly mediates various biological processes, including tumorigenesis [13, 14]. The m^6^A methyltransferase complex contains several enzymes (namely METTL3, METTL14, and WTAP), which are known as “writers,” of which METTL3 (methyltransferase-like 3) is the most important because of its pivotal roles in regulating gene expression by influencing RNA stability, mRNA degradation, and translation [15, 16]. In contrast, FTO (α-ketoglutarate-dependent dioxygenase) and ALKBH5 (AlkB homolog 5, RNA demethylase) have the potential to remove m^6^A from mRNA by functioning as “erasers” [17, 18]. In addition, YTH family proteins, IGF2BPs, and eIF3s have been shown to act as m^6^A “readers” to affect mRNA stability and translation to mediate pleiotropic downstream effects [15, 19, 20]. Emerging evidence has illustrated that abnormal m^6^A mRNA methylation is intimately involved in the onset and progression of human cancers, including leukemia [21], brain cancer [22], breast cancer [23], liver cancer [24], and endometrial cancer [25]. Additionally, multiple m^6^A-regulated proteins and their target mRNAs display varying expression profiles in cancers, suggesting that m^6^A modification plays an oncogenic or tumor-suppressive role under different genomic backgrounds or in different tumor subtypes [26, 27]. Therefore, it is necessary to elucidate the underlying mechanism in detail to reveal the exact biological process and effects of m^6^A modification in tumorigenesis.

We previously confirmed that low FBXW7 expression in patients with EGFR inhibitor-resistant NSCLC is correlated with upregulated Mcl-1 expression. FBXW7 reactivation reduced Mcl-1 expression, in turn, increasing the tumor metastatic, invasive, and drug-resistant capacities of lung cancer by depredating specific substrates [28, 29]. In this study, we investigated the functions of FBXW7 and its potential associated regulators in lung cancer tumorigenesis. We found that METTL3 mediated m^6^A-modification of the FBXW7 mRNA coding sequence (CDS), promoting FBXW7 translation at the posttranscriptional level. Our results reveal an anticancer role and novel regulatory mechanisms for m^6^A-modified FBXW7 in LUAD, which may be applicable for molecular diagnosis and targeted therapy.

## Methods

### Ethics statement

This study was approved by the Ethics Committee of the Fourth Military Medical University. All animal experiments were carried out in strict accordance with the Guide for the Care and Use of Laboratory Animals published by the US National Institutes of Health.

### Cell culture and reagents

The human LUAD cell lines HCC827 and PC9 have been fully described. All cell lines were obtained between 2016 and 2019 and validated by short tandem repeat analysis, tested for mycoplasma contamination within the last six months, and used at passage numbers of < 10. HCC827 and PC9 cells were cultured in RPMI 1640 (Corning, Inc., Corning, NY, USA).

All compounds were purchased from Selleck Chemicals (Houston, TX, USA), except for D-luciferin (Promega, Madison, WI, USA), cycloheximide (Sigma, St. Louis, MO, USA), and puromycin (Sigma), and were dissolved in dimethyl sulfoxide for cell culture.

### Tumor samples and immunohistochemical (IHC) analysis

LUAD tissues and surrounding tissues were purchased from Shanghai Outdo Biotech (Shanghai, China) and collected from Tangdu Hospital (Xian, China). For IHC staining, the sections were deparaffinized in xylene, rehydrated using graded ethanol, and incubated with 0.1% sodium citrate buffer (pH 6.0) for 20 min at 95 °C for antigen retrieval. After endogenous peroxidase activity had been quenched with 3% H_2_O_2_·dH_2_O and nonspecific binding had been blocked with 1% bovine serum albumin buffer, the sections were incubated overnight at 4 °C with anti-METTL3 (ab195352, 1:1000, Abcam, Cambridge, UK) or anti-FBW7α (A301–720, 1:1000, Bethyl Laboratories, Montgomery, TX, USA) antibodies. Following several washes, the sections were treated with horseradish peroxidase-conjugated secondary antibodies for 30 min at room temperature and stained with 0.05% 3, 3- diaminobenzidine tetrahydrochloride. The slides were then photographed using a virtual slide microscope (Olympus VS120, Tokyo, Japan), and the images were analyzed with Image-Pro Plus 6.0 software (Media Cybernetics, Silver Spring, MD, USA). IHC sections were analyzed by two independent investigators.

### Molecular biology

FBXW7 and METTL3 constructs were prepared using the pcDNA 3.1 vector (Invitrogen, Carlsbad, CA, USA).

### RNA isolation and reverse transcription-quantitative polymerase chain reaction (RT-qPCR)

Total RNA was isolated using RNeasy reagents (Qiagen, Hilden, Germany), and real-time PCR (qRT-PCR) was performed using an iCycler Real-Time System (Bio-Rad Laboratories, Hercules, CA, USA) with a SYBR Premix EX Tag Mastermix kit (TaKaRa, Shiga, Japan) according to the manufacturer’s instructions. Glyceraldehyde-3-phosphate dehydrogenase was used as an internal reference for gene expression. The relative mRNA expression of target genes was expressed using the 2^-ΔΔCt^ method. Experiments were repeated three times [30]. The same method was used for mRNA detection in subsequent cell experiments. The primers used are listed in Additional file 2: Table S1.

### Gene overexpression and knockdown

Lentiviral vectors containing METTL3 shRNA were purchased from Genchem (Shanghai, China). Cells were seeded in a 6-cm dish at a density of 2.5 × 10^6^ cells, allowed to attach for 12 h, and transfected with overexpression plasmid (2 μg) or siRNA (1.5 μg) targeting each gene using Lipofectamine® 2000 reagent (Invitrogen) according to the manufacturer’s instructions. The knockdown siRNA sequences are listed in Additional file 2: Table S2.

### Western blot analysis

Cells were washed with ice-cold phosphate-buffered saline, scraped from the culture dishes, centrifuged at 12,000 rpm at 4 °C for 15 min, and then lysed in radioimmunoprecipitation assay lysis buffer (Thermo Fisher Scientific, Waltham, MA, USA) before being centrifuged at 15,000×*g* for 20 min at 4 °C. The protein concentration was determined using a BCA protein assay kit (Thermo Fisher Scientific). Proteins were then separated by SDS-PAGE, transferred to a polyvinylidene fluoride membrane (Millipore, Billerica, MA, USA), and blocked with 5% non-fat milk. Next, the membranes were incubated with antibodies or conjugated to horseradish peroxidase for 2 h at room temperature as a control. Proteins were visualized using electrochemiluminescence reagents (GE Healthcare Life Sciences, Little Chalfont, UK) and detected using a LAS-4000 imaging system (Fujifilm, Tokyo, Japan). Image density was quantified using ImageJ analysis software (NIH, Bethesda, MD, USA). The antibodies used are listed in Additional file 2: Table S3.

### Half-life analysis

After gene manipulation, 20 μg/mL of cycloheximide (CHX; Sigma, final concentration 100 μg/mL) was added to the cell medium, and the cells were harvested at the indicated time points, lysed, and FBXW7 expression was measured by western blot analysis.

### RNA-binding protein immunoprecipitation (RIP)

RIP assays were performed using a Magna RIPTM RNA-Binding Protein Immunoprecipitation Kit (Millipore) according to the manufacturer’s protocol. Briefly, the cells were collected and lysed in a complete radioimmunoprecipitation assay buffer containing a protease inhibitor cocktail and RNase inhibitor. Antibodies (5 μg) were pre-bound to Protein A/G magnetic beads in immunoprecipitation buffer (20 mM Tris-HCl pH 7.5, 140 mM NaCl, 0.05% TritonX-100) for 2 h and then incubated with 100 μL of cell lysate overnight at 4 °C with rotation. RNA was eluted from the beads by incubation with 400 μL of elution buffer for 2 h, precipitated with ethanol, and dissolved in RNase-free water. The enrichment of certain fragments was determined by real-time PCR.

### m^6^A RNA immunoprecipitation (MeRIP) assay

MeRIP assays were performed using a Magna MeRIP m^6^A Kit (Millipore) according to the manufacturer’s instructions. Briefly, RNAs were chemically fragmented to ~ 100 nucleotides and incubated with magnetic beads conjugated to m^6^A antibodies (Abcam) for immunoprecipitation. The enrichment of m^6^A-containing mRNA was analyzed by qRT-PCR and normalized to the input.

### Dual-luciferase reporter assay

Cells were seeded into each well of a 24-well plate and co-transfected with vectors according to the Lipofectamine®2000 (Invitrogen) protocol. After 48 h, luciferase activity was measured using a Dual-Glo Luciferase Assay system (Promega) according to the manufacturer’s instructions. Relative luciferase activity was assessed using a SYNERGY microplate reader (BioTek, Winooski, VT, USA). Firefly luciferase (F-luc) activity was normalized using Renilla luciferase to evaluate reporter translation efficiency.

### Cell apoptosis analysis

Cells were incubated with Annexin V and propidium iodide (PI) according to the manufacturer’s instructions to analyze apoptosis. After 30 min, the cells were analyzed by fluorescence-activated cell sorting using a flow cytometer (BD Biosciences, Franklin Lakes, NJ, USA). Annexin V-FITC-staining indicated early apoptosis, whereas cells with double-positive Annexin V-FITC and PI signals were pooled for analysis.

### Cell proliferation assay

Cell proliferation was assessed using a Cell Counting Kit-8 (CCK-8; Dojindo Laboratories, Kumamoto, Japan) according to the manufacturer’s instructions. Briefly, the cells were seeded in 96-well plates (5 × 10^3^ cells/well), and then the medium was discarded. Next, a diluted CCK-8 solution (10% of total volume) was added at the indicated time points and incubated for a further 1.5 h. Absorbance was measured at 570 nm with a spectrophotometer (Tecan, Männedorf, Switzerland). Experiments were conducted in triplicate.

### In vivo tumor formation assay

A total of 20 BALB/c nude mice (4–6 weeks old; weighing 17–20 g) were purchased from Cavens Laboratory Animal Co., Ltd. (Changzhou, China) and housed in a specific pathogen-free environment. The mice were randomly divided into groups before subcutaneous injection with cells (10^7^) suspended in 200 μL of phosphate-buffered saline. Tumors were measured on day 7 and then once every 7 days until 28 days after injection. Tumor volume was calculated using the following formula: V (mm^3^) = 0.5 × length (mm) × width^2^ (mm^2^). At 4 weeks post-injection, the mice were sacrificed, and their tumors were isolated and weighed.

### Statistical analysis

Statistical analyses were performed using Image-Pro Plus 6.0, ImageJ, and GraphPad Prism 8.0 (GraphPad, Inc., La Jolla, CA, USA) software. The mean values and standard deviation were calculated for continuous variables. One-way analysis of variance was used to determine variance among multiple groups. Survival curves were assessed using the Kaplan-Meier method and log-rank tests. A *p*-value < 0.05 was considered statistically significant.

## Results

### m^6^A methylation of FBXW7 mRNA by METTL3

**Fig. 1:**
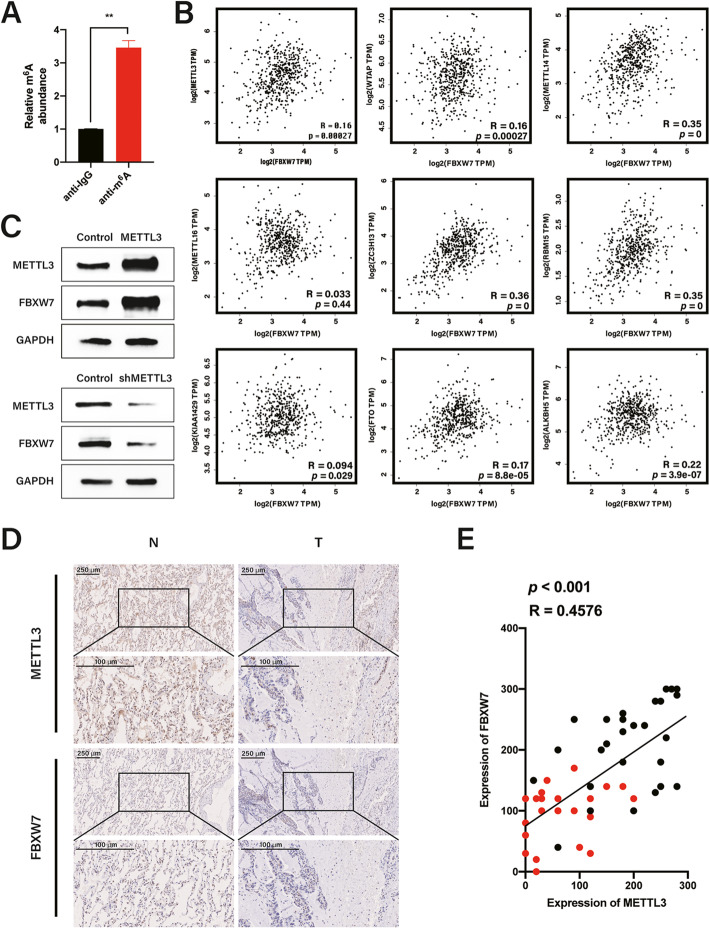
m^6^A methylation of FBXW7 mRNA by METTL3. **a** m^6^A abundance on FBXW7 mRNA in HCC827 cells detected by MeRIP. **b** Gene correlation analysis of FBXW7 and m^6^A enzymes by GEPIA using Spearman statistics in lung adenocarcinoma (LUAD). **c** Western blotting of FBXW7 and METTL3 in HCC827 cells with METTL3 overexpression or knockdown. **d** Representative immunohistochemical staining for METTL3 and FBXW7 in LUAD and adjacent normal tissue (scale bar: 250 and 100 μm). **e** Correlation analysis between FBXW7 and METTL3 in twenty-eight pairs of normal (N) and LUAD tumor (T) samples. Correlation chart drawn in GraphPad Prism Version 8.4.0. Red: tumor; Black: normal. *r* = 0.4576, *p* < 0.001. (Pearson’s correlation analysis). Bar = mean ± SD. **p < 0.01

Substantial evidence has confirmed that FBXW7 plays a negative role in the pathogenesis of lung cancers. To explore the function of m^6^A methylation in the epigenetic regulation of FBXW7, we examined whether FBXW7 mRNA was m^6^A-methylated. MeRIP-qPCR indicated that FBXW7 mRNA contained abundant m^6^A modifications in HCC827 and PC9 cells (Fig. 1a, Additional file 1: Fig. S1A). To confirm the catalytic proteins that may be involved in m^6^A modification of FBXW7 mRNA, we compared and analyzed the correlations between FBXW7 and demethylases or methyltransferases from TCGA (https://portal.gdc.cancer.gov/) [31]. Interestingly, FXBW7 expression exhibited a positive correlation with all m^6^A modification system-related proteins except for METTL16 in LUAD (Fig. 1b); therefore, we predicted that the loss of FBXW7 was related to deregulation of m^6^A-related enzymes in LUAD, particularly the aberrant expression of METTL3, which is a key “writer” that installs m^6^A on RNAs.

Simultaneously, we constructed METTL3 overexpression or knockdown systems using lentiviruses to study the function of METTL3 in LUAD (Additional file 1: Fig. S1B–D) and its effect on FBXW7 protein expression. We found that METTL3 overexpression in HCC827 and PC9 cells upregulated FBXW7 protein levels (Fig. 1c upper panels, Additional file 1: Fig. S1D, left panels), whereas METTL3 depletion significantly reduced FBXW7 protein expression (Fig. 1c, bottom panels, Additional file 1: Fig. S1D, right panels).

We also analyzed FBXW7 and METTL3 protein expression in twenty-eight paired LUAD and normal tissues, with IHC staining revealing that METTL3 and FBXW7 protein levels were higher in normal adjacent lung tissues than in LUAD tissues. Consistently, FBXW7 was expressed at lower levels in LUAD samples with low METTL3 expression (Fig. 1d). After standardization, the relationship between FBXW7 and METTL3 was plotted as a scatter diagram (Fig. 1e), which revealed that decreased FBXW7 protein expression was accompanied by decreased METTL3 expression and indicated a strong correlation between FBW7 and the m^6^A “writer.” Together, these results demonstrate that METTL3 mediates m^6^A modification in FBXW7 mRNA.

### Correlation between METTL3 downregulation and poor prognosis in patients with LUAD

**Fig. 2:**
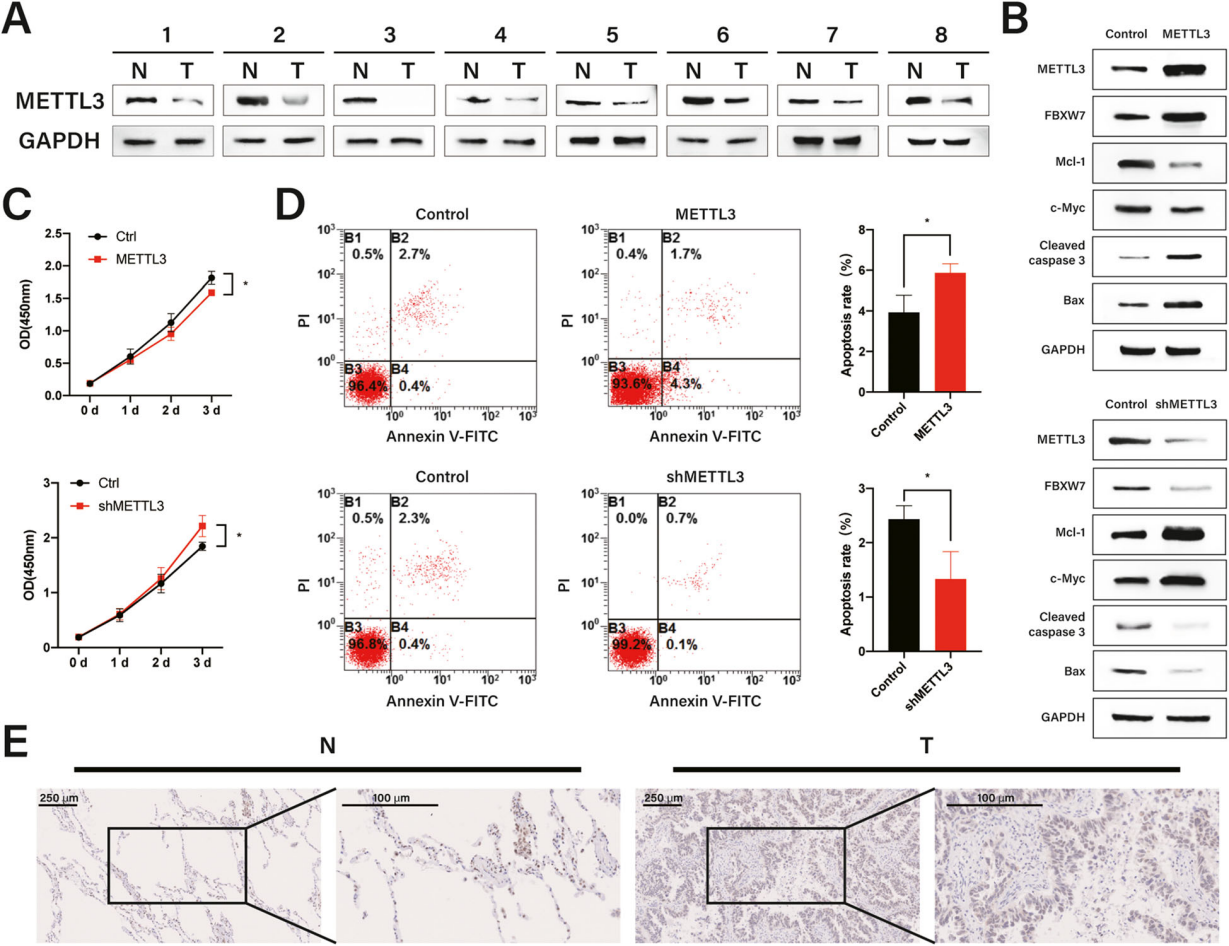
Correlation between METTL3 downregulation and unfavorable prognosis in patients with lung adenocarcinoma (LUAD). **a** Relative METTL3 protein levels in paired tissues from eight patients with LUAD. **b** Western blotting of Mcl-1, c-Myc, cleaved caspase 3, Bax, METTL3, and FBXW7 protein expression in HCC827 cells with METTL3 overexpression or depletion. **c–d** METTL3 overexpression or depletion affected HCC827 cell apoptosis and proliferation. **e** Representative immunohistochemical staining for METTL3 in LUAD and paired normal tissues from one patient. Bar = mean ± SD. *p < 0.05

Recent studies have suggested that the m^6^A modification of many proteins plays a critical role in the development of various cancers; however, the role of METTL3 in LUAD remained unclear. We analyzed METTL3 protein expression in eight paired LUAD and normal tissues and found that METTL3 was remarkably downregulated in LUAD tissues (Fig. 2a). To identify direct targets of METTL3, we investigated changes in protein expression following METTL3 overexpression or knockdown in HCC827 and PC9 cells. We found that METTL3 overexpression increased the protein levels of apoptosis molecules (cleaved caspase 3 and Bax) and decreased the protein levels of Mcl-1 and c-Myc, which are classical FBXW7 target genes. In contrast, METTL3 depletion in HCC827 and PC9 cells had the opposite effects (Fig. 2b, Additional file 1: Fig. S2A).

To investigate the contribution of m^6^A toward LUAD development and progression, we focused on apoptosis and proliferation, which play key roles in the progression of various cancers. Although METTL3 depletion reduced apoptosis and promoted HCC827 and PC9 cell proliferation (Fig. 2c–d, Additional file 1: Fig. S2B–C, upper panels), its overexpression strongly enhanced apoptosis and impeded proliferation compared to in control cells (Fig. 2c–d, Additional file 1: Fig. S2B-C, bottom panels). In addition, we evaluated the clinical relevance of METTL3 protein levels in LUAD using IHC staining in eight paired patient samples. METTL3 expression was weak or undetectable in most LUAD samples but was moderate or high in most para-tumor controls (Fig. 2e), suggesting that METTL3 reduces LUAD tumorigenesis.

### FBXW7 overexpression rescues the anti-tumor phenotype impaired by METTL3 knockdown in vitro

**Fig. 3:**
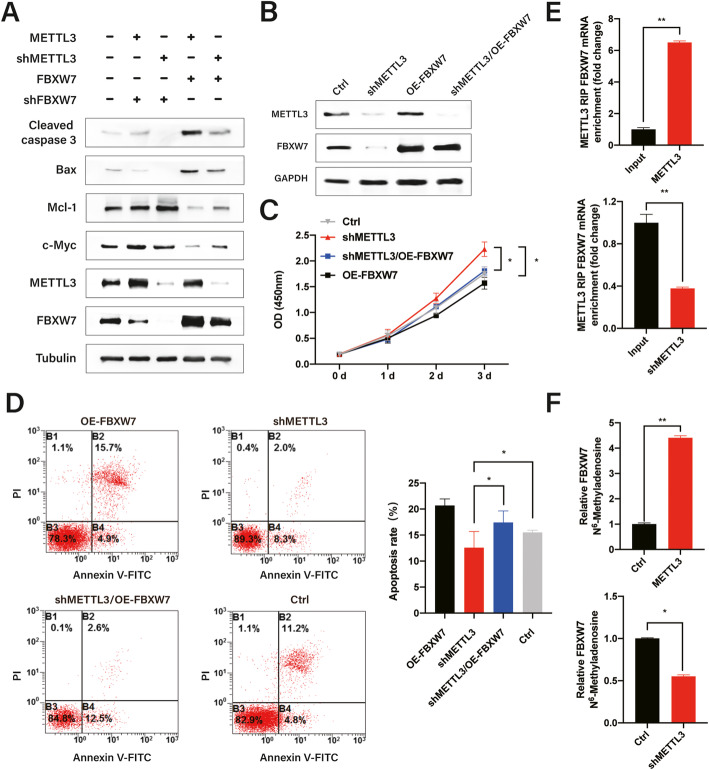
FBXW7 overexpression rescues the anti-tumor phenotype impaired by METTL3 knockdown in vitro. **a** Relative protein levels of Mcl-1, c-Myc, cleaved caspase 3, Bax, METTL3, and FBXW7 were detected in HCC827 cells with the indicated plasmids. **b** METTL3 and FBXW7 expression were analyzed by western blot analysis in HCC827 cells with the indicated plasmids. **c–d** Effects of FBXW7 overexpression in shMETTL3 HCC827 cells. FBXW7 partially restored apoptosis and the inhibition of proliferation in HCC827 cells, which were reduced by METTL3 knockdown. **e** Radioimmunoprecipitation assays demonstrated the association between METTL3 and FBXW7 mRNA in HCC827 cells. **f** FBXW7 m^6^A modification levels in HCC827 cells determined using MeRIP-qPCR. Bar = mean ± SD. *p < 0.05, **p < 0.01

To verify whether the impaired tumorigenesis phenotype in METTL3-depleted cells depended on FBXW7 reduction, we overexpressed FBXW7 in both HCC827 and PC9 subclones with METTL3 knockdown. Western blotting revealed that FBXW7 overexpression abrogated the increased expression of its downstream targets (Mcl-1 and c-Myc) and decreased the expression of apoptosis molecules (cleaved caspase 3 and Bax) in cells with METTL3 knockdown (Fig. 3a, Additional file 1: Fig. S3A).

To validate whether FBXW7 contributes to the anti-tumor phenotype regulated by METTL3, we generated stable cells expressing the indicated genes (Fig. 3b, Additional file 1: Fig. S3B) to determine whether FBXW7 overexpression could rescue the effects of METTL3 knockdown on the biological behavior of HCC827 or PC9 cells. As shown in Fig. 3c–d, FBXW7 overexpression partially reversed the effects of METTL3 depletion on the apoptosis and proliferation of HCC827 cells and reversed the phenotype caused by METTL3 deficiency in PC9 cells (Additional file 1: Fig. S3C–D). Collectively, these results indicate that FBXW7 overexpression rescues the impaired anti-tumor phenotype caused by METTL3 knockdown.

In addition, quantitative RIP assays suggested that FBXW7 mRNA interacts with METTL3 in HCC827 and PC9 cells (Fig. 3e, Additional file 1: Fig. S3E). To verify that m^6^A-modified FBXW7 is regulated by METTL3, we performed MeRIP-qPCR experiments in HCC827 and PC9 cells with METTL3 overexpression or knockdown. METTL3 overexpression significantly promoted m^6^A modification in FBXW7 mRNA. However, METTL3 depletion had the opposite effect in HCC827 and PC9 cells (Fig. 3f, Additional file 1: Fig. S3F).

### METTL3 triggers enhanced FBXW7 mRNA translation

**Fig. 4:**
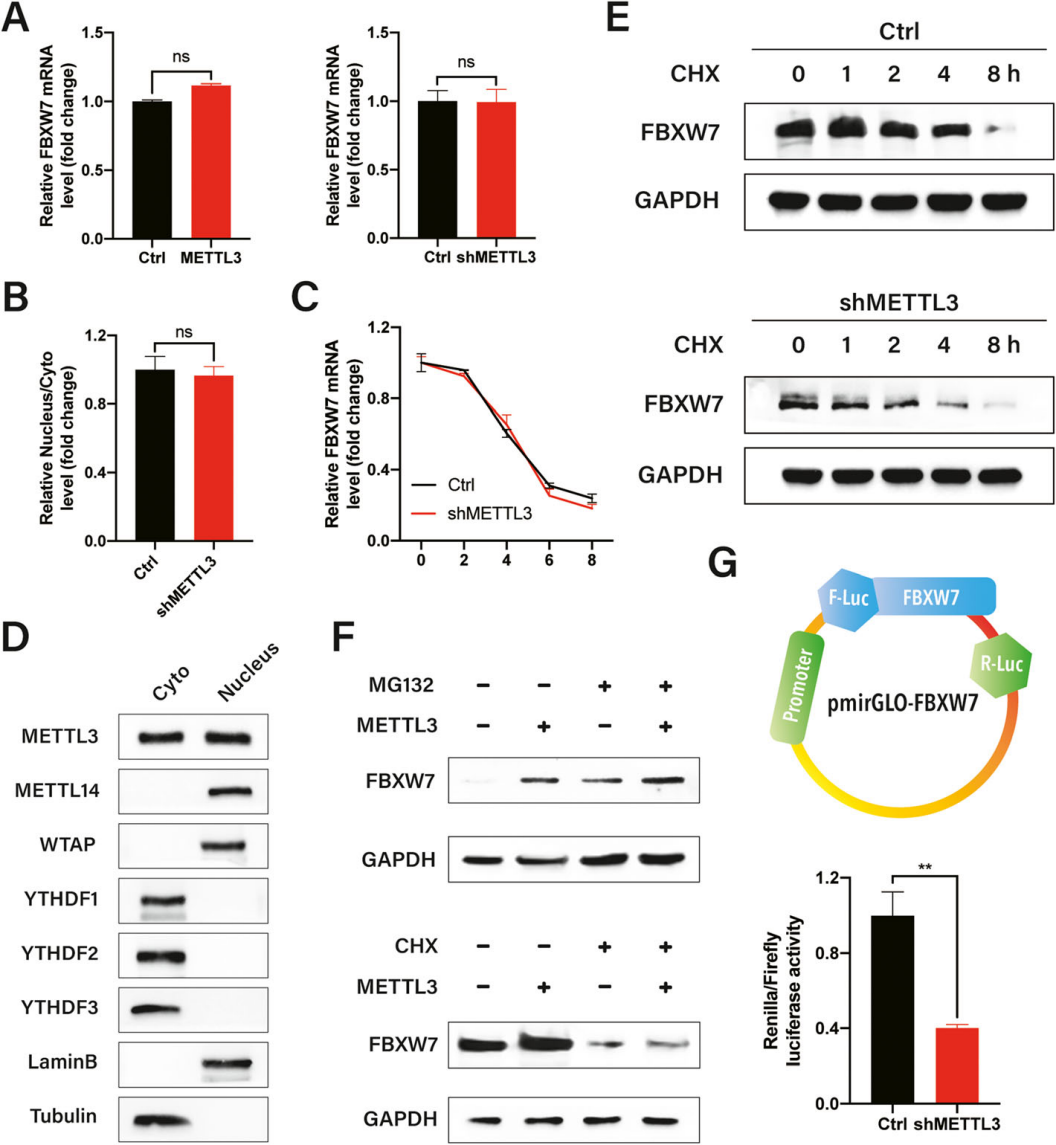
METTL3 triggered enhanced FBXW7 mRNA translation. **a** FBXW7 mRNA was analyzed by RT-PCR in HCC827 cells with METTL3 overexpression or depletion. **b** Nuclear and cytoplasmic FBXW7 mRNAs were extracted separately. **c** FBXW7 mRNA was analyzed in HCC827 cells transfected with METTL3 control or shRNA plasmids after ActD treatment. **d** Western blot analysis of the cytoplasmic and nuclear fractions of HCC827 cells using polyclonal antibodies against METTL3, METTL14, WTAP, YTHDF1/2/3, LaminB (a nuclear protein), and α-tubulin (a cytoplasmic protein). **e** FBXW7 protein expression was determined by western blot analysis in HCC827 cells treated with CHX to block protein synthesis. **f** HCC827 cells were pretreated with CHX or MG-132 and then treated with control vectors or METTL3 plasmids. **g** Firefly (F-Luc) values were normalized against Renilla luciferase levels, and FBXW7 translation efficiency was calculated for the pmirGLO-FBXW7 reporter relative to pmirGLO in METTL3 knockdown and control HCC827 cells. Bar = mean ± SD. n.s., not significant; **p < 0.01

Next, we investigated the mechanisms via which m^6^A modification affects FBXW7 expression in LUAD. First, evaluation of FBXW7 mRNA levels revealed no major differences in shMETTL3 or overexpressing cells (Fig. 4a). To verify whether METTL3 affected the subcellular FBXW7 mRNA distribution, we isolated and analyzed cytoplasmic and nuclear RNAs (Additional file 1: Fig. S4A); however, no notable discrepancy was observed in the subcellular localization of FBXW7 mRNA between METTL3 knockdown and control HCC827 cells (Fig. 4b). Similarly, no dramatic differences in the half-life of FBXW7 mRNA were detected in METTL3 knockdown and control HCC827 cells, as measured using actinomycin D (Fig. 4c). Consequently, protein stability or translation efficiency may be responsible for the reduced FBXW7 protein expression in shMETTL3 HCC827 cells.

To test this assumption and validate whether METTL3 is engaged in translational control, we explored whether METTL3 was localized in the cytoplasmic fractions where the translation is initiated and occurs. Apart from nuclear localization where m^6^A modification occurs, METTL3 was expressed in the cell cytoplasm; however, other known METTL3-interacting proteins, including the m^6^A methyltransferase METTL14 and cofactor WTAP, displayed pronounced nuclear localization (Fig. 4d). To assess the potential effects of METTL3 on FBXW7 protein stability, we blocked translation using CHX and measured FBXW7 degradation in METTL3 knockdown and control HCC827 cells. The western blot analysis results revealed that the half-life of FBXW7 proteins was similar in METTL3 knockdown and control HCC827 cells (Fig. 4e), excluding the possibility that m^6^A -induced FBXW7 expression was associated with protein stability. HCC827 cells were also pretreated with the proteasome inhibitor MG132 or translation inhibitor CHX and then transfected with METTL3 overexpression plasmids. Interestingly, impairing METTL3 induced FBXW7 expression in HCC827 cells in the presence of CHX but not MG-132 (Fig. 4f). The PmirGLO-FBXW7-CDS luciferase reporter was generated by ligating the FBXW7 CDS to a multiple cloning site (Fig. 4g upper panel), and the subsequent assay indicated that the translational efficiency of FBXW7 was significantly lower in shMETTL3 cells than in the control group (Fig. 4g, bottom panel). Together, these results suggest that m^6^A mRNA modification regulates FBXW7 translation.

### m^6^A methylated motifs in the CDS of FBXW7 promote its translation

**Fig. 5:**
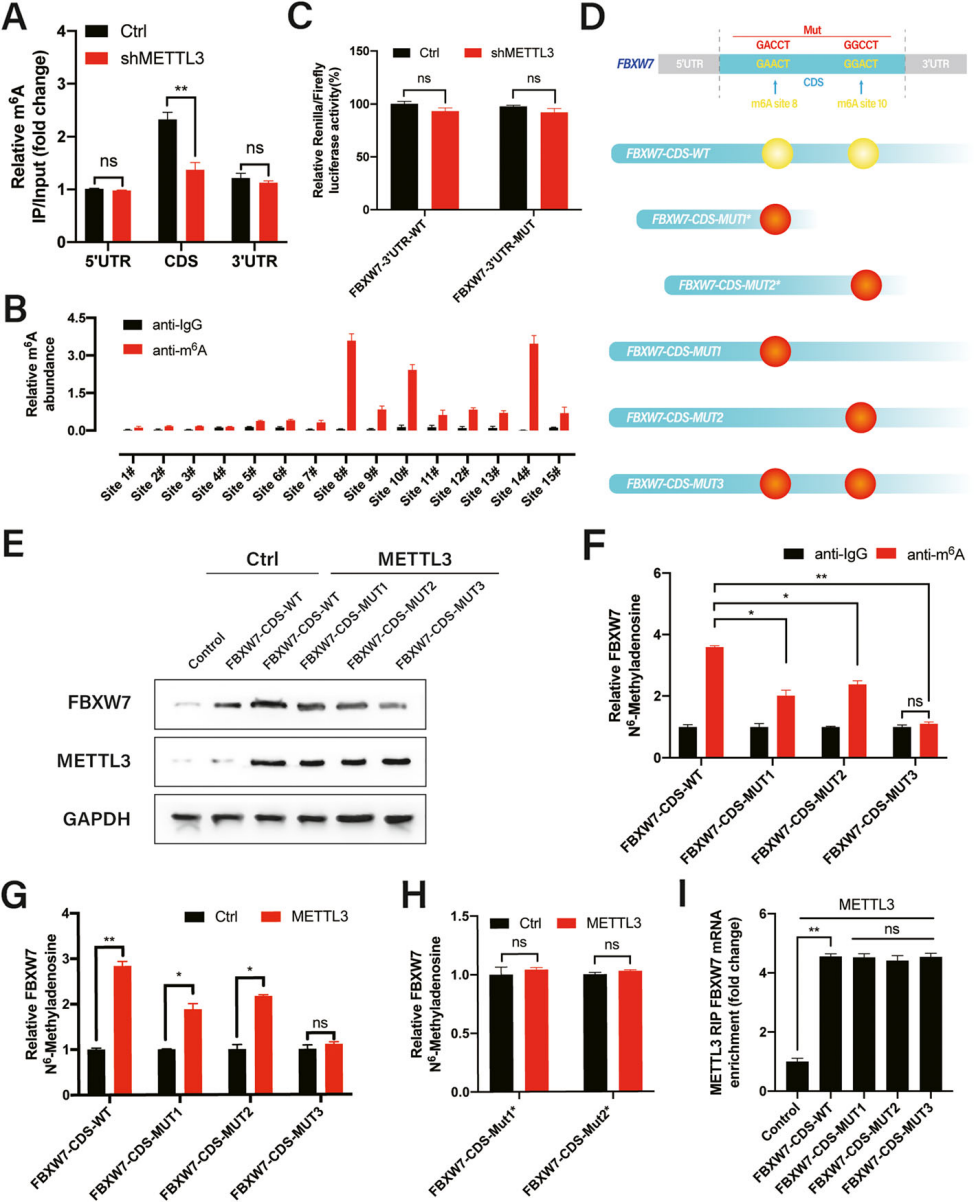
m^6^A methylated motifs in the CDS promote FBXW7 translation. **a** m^6^A levels of fragmented RNA detected in wild-type or shMETTL3 HCC827 cells using MeRIP-qPCR. **b** m^6^A levels of predicted m^6^A sites analyzed by MeRIP-qPCR. Background noise was removed by normalizing samples to non-crosslinked inputs. Anti-IgG antibodies were used as a control. **c** Control shRNA and METTL3 knockdown HCC827 cells were transfected with or without PmirGLO-FBXW7–3’UTR-WT or PmirGLO-FBXW7–3’UTR-MUT. **d** Predicted m^6^A sites (yellow) and synonymous mutations (red) in the CDS sequence of FBXW7 mRNA. **e** Western blot analysis of FBXW7 expression in HCC827 cells co-expressing exogenous METTL3 and FBXW7-CDS-WT or FBXW7-CDS-MUTs. **f** qPCR analysis of immunoprecipitated m^6^A in HCC827 cells transfected with the indicated plasmids using FBXW7 PCR primers. **g–i** MeRIP-qPCR analysis of FBXW7 mRNA in HCC827 cells transfected with the indicated genes. Bar = mean ± SD. n.s., not significant; *p < 0.05, **p < 0.01

Next, we attempted to elucidate the molecular mechanisms via which METTL3 promotes the translational efficiency of FBXW7. Fragmented RNA isolated from HCC827 cells was immunoprecipitated using m^6^A antibodies to investigate the distribution of m^6^A methylation in FBXW7 mRNA. The highest levels of m^6^A methylation were observed in the CDS, followed by the 3′ untranslated region (UTR) and 5′UTR (Fig. 5a). Accordingly, decreased m^6^A enrichment was observed in the FBXW7 CDS in shMETTL3 cells, indicating that m^6^A modification is more dynamic in the CDS than in the 3′UTR region. Moreover, we predicted possible m^6^A modification sites in the FBXW7 sequence using the sequence-based RNA adenosine methylation site predictor (SRAMP) [32], which valued each identified m^6^A site by assigning a predictive score (Additional file 1: Fig. S5, Additional file 2: Table S4). The FBXW7 genome contained 26 potential m^6^A sites predicted by SRAMPS, of which four had very high confidence at positions 2066, 2111, 2264, and 3190. To verify these predicted m^6^A sites, we designed specific primers to amplify 15 sites, which were merged into one region of less than 200 base pairs (bp), and MeRIP-qPCR was performed. After normalizing m^6^A-precipitated signals to the input, only three m^6^A sites (sites 8#, 10#, and 14#) showed elevated levels, indicating that the m^6^A sequence exists in these sites (Fig. 5b). Subsequently, we identified sites #11, #16, and #23 in cells transfected with the indicated mutant plasmids (RRACH → RRCCH) using MeRIP (Additional file 1: Fig. S4B).

To explore the potential roles of m^6^A methylation in the FBXW7 3′UTR region, which demonstrated m^6^A methylation at site #23, we performed a luciferase assay in HCC827 cells using reporters containing FBXW7–3′UTR-WT or -MUT (site #23: AGAC to AGCC.). Compared to FBXW7–3′UTR-WT, the translational activity of FBXW7–3′UTR-MUT was similar between the control and shMETTL3 group, and METTL3 overexpression did not alter the luciferase activity of FBXW7–3′UTR-WT or -MUT in HCC827 cells (Fig. 5c). Together, these results indicate that the m^6^A modification, which promotes FBXW7 translation, does not correlate with m^6^A methylation levels at sites in the 3′UTR.

Therefore, we examined whether m^6^A methylation in the CDS promotes the translation of FBXW7. First, we constructed an FBXW7 CDS expression plasmid and mutated the m^6^A motif in the coding region of FBXW7, as follows: FBXW7-CDS-MUTs*: FBXW7-CDS-MUT1* and FBXW7-CDS-MUT2* (containing only one predicted m^6^A site that was mutated to create FBXW7-CDS-MUT1/2); FBXW7-CDS-MUTs series: FBXW7-CDS-MUT1, FBXW7-CDS-MUT2 (containing three putative m^6^A sites of which only one was mutated to create FBXW7-CDS-MUT1/2), and FBXW7-CDS-MUT3 (containing two potential m^6^A sites, which were both mutated to form FBXW7-CDS-MUT3; Fig. 5d). FBXW7 protein expression was lower in cells co-transfected with METTL3 and FBXW7-CDS-MUTs than in the METTL3 and FBXW7-CDS-WT group (Fig. 5e). We also performed MeRIP-qPCR to examine the level of m^6^A modification, which showed that the FBXW7-CDS-WT had high levels of m^6^A modification, whereas FBXW7 MUTs (MUT1, MUT2, and MUT3) contained little or no m^6^A in HCC827 cells with endogenous METTL3 expression (Fig. 5f). METTL3 overexpression significantly increased the abundance of m^6^A in FBXW7-CDS-WT/MUTs compared to the control vector of METTL3; however, this effect was inhibited in FBXW7 MUTs compared to in the FBXW7-CDS-WT because of the mutated m^6^A site. Remarkably, the m 6 A modification of FBXW7 MUT3, containing two m 6 A site mutation, was not elevated in METTL3 overexpression and control HCC827 cells (Fig. 5g). Compared to the control group, m^6^A modification was not enhanced in FBXW7-MUT1/2 in METTL3-overexpressing cells. Notably, the levels of transfected FBXW7 MUTs* were unchanged in METTL3-overexpressing compared to the control METTL3 vector due to only having one mutated m^6^A site (Fig. 5h), suggesting that the putative sites in FBXW7 mRNA are m^6^A methylated by METTL3. Furthermore, FBXW7-CDS-WT and FBXW7 MUT mRNAs interacted with METTL3, revealing that the mutated sites did not interrupt direct interaction between METTL3 and FBXW7 MUT mRNAs (Fig. 5i). Therefore, these results exclude the possibility that mutation interfered with the interaction between FBXW7 MUTS and METTL3. Together, our data indicate that METTL3 site-specific m^6^A modifications in the FBXW7 CDS promote FBXW7 translation.

### FBXW7 overexpression rescued tumor growth inhibition impeded by METTL3 knockdown in vivo

**Fig. 6:**
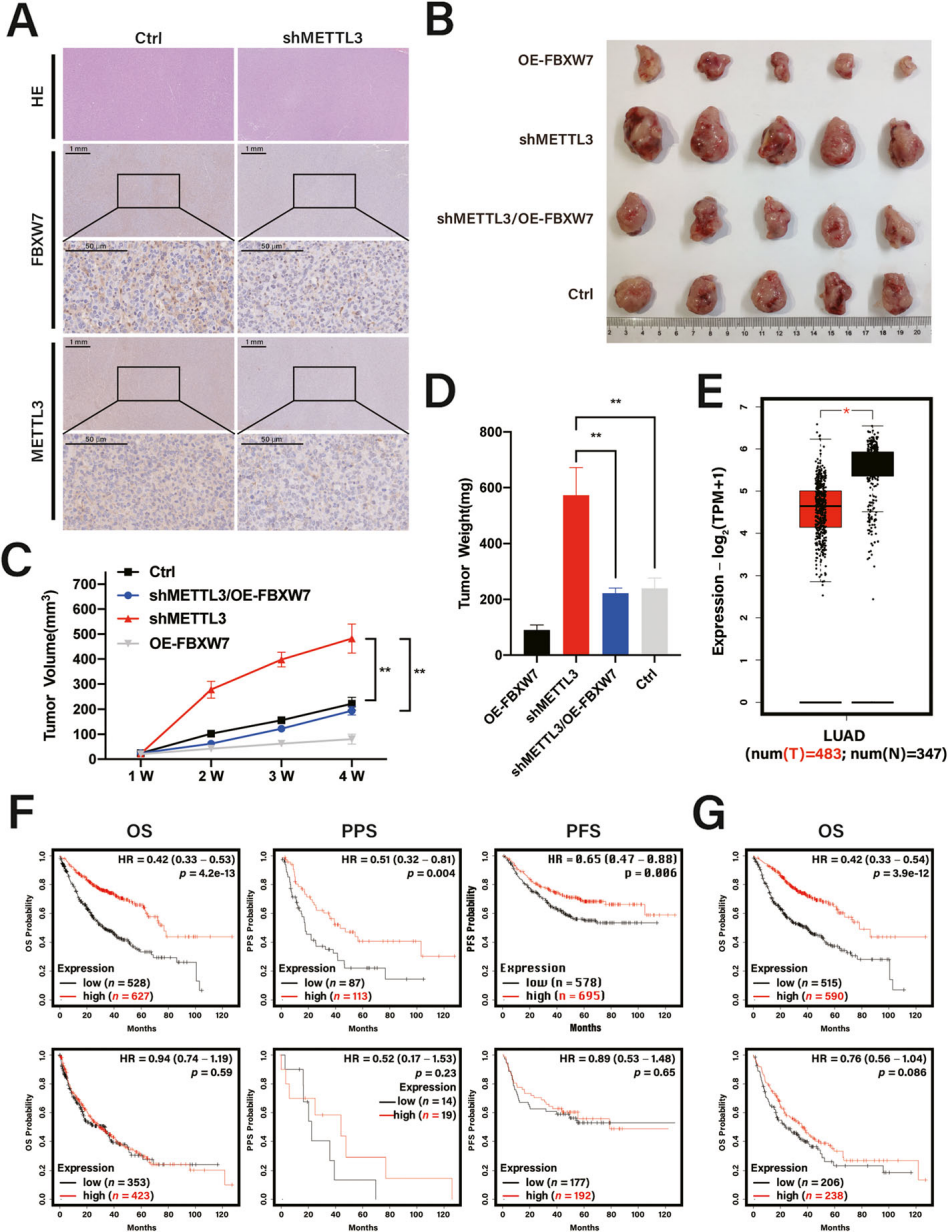
FBXW7 overexpression rescues tumor growth inhibition impeded by METTL3 knockdown in vivo. **a** Representative IHC staining images of METTL3 and FBXW7 expression in mouse tumors. **b** Tumors from nude mice. **c** Volume of HCC827 cell tumors xenografted with the indicated plasmids in nude mice measured at regular intervals. **d** Weight of HCC827 cell tumors xenografted with the indicated plasmids in nude mice. **e** Results based on TCGA and GEPIA database (http://gepia.cancer-pku.cn/index) showed the expression level of METTL3 in patients with lung adenocarcinoma (LUAD). **f** Kaplan-Meier analysis of METTL3 for overall survival, post-progression survival, and progression-free survival in LUAD (upper panels) and lung squamous cell carcinoma (LUSC) (bottom panels) patients. **g** Kaplan-Meier analysis of FBXW7 for OS in LUAD (upper panels) and LUSC (bottom panels) patients. Bar = mean ± SD. ^*^p < 0.05; **p < 0.01

To validate the effect of METTL3 on tumor growth in vivo, we conducted a xenograft tumor assay by subcutaneously injecting nude mice with HCC827 Ctrl, shMETTL3, OE-FBXW7, and shMETTL3/OE-FBXW7 cells. Significantly more tumors were derived from shMETTL3 HCC827 cells than from HCC827 Ctrl cells; however, FBXW7 overexpression attenuated the tumor-promoting effect of shMETTL3 HCC827 cells in vivo (Fig. 6b). We found that the shMETTL3/OE-FBXW7 groups displayed smaller tumors (Fig. 6c) and slower tumor growth (Fig. 6d) than the shMETTL3 group (*p* < 0.01), whereas peritumoral tissues displayed high FBXW7 levels and a remarkable reduction in tumor size. Additionally, METTL3 exhibited a similar expression pattern to FBXW7 (Fig. 6a), indicating a positive correlation between FBXW7 and METTL3 at the protein level.

Low METTL3 expression in LUAD was validated by Gene Expression Profiling Interactive Analysis (GEPIA, http://gepia.cancer-pku.cn/index.html) [33] (Fig. 6e) and the prognostic correlation between METTL3 mRNA expression and the survival of patients with LUAD was evaluated using publicly available datasets. Kaplan-Meier curves revealed that patients with LUAD with higher METTL3 expression had better overall survival (*n* = 1927, *p* = 4.2 × 10^− 13^), post-progression survival (*n* = 344, *p* = 0.004), and progression-free survival (*n* = 982, *p* = 0.006; Fig. 6f, upper panels). Interestingly, this effect was markedly more significant in patients with LUAD than in those with lung squamous cell carcinoma (LUSC) (Fig. 6f, lower panels), consistent with the overall survival analysis of FBXW7 (Fig. 6g). Taken together, these data suggest that the METTL3-FBXW7 axis contributes to inhibition of tumor growth in vivo and that targeting m^6^A-modified FBXW7 is a promising strategy for overcoming tumorigenesis in LUAD.

## Discussion

FBXW7 is a key substrate recognition subunit of the SCF complex that acts as a tumor suppressor gene by controlling the proteasome-mediated degradation of oncoproteins, such as c-MYC, MCL1, Notch1/4, cyclin E, and mTOR [4, 5]. Several studies have shown that the loss-of-function mutation of FBXW7 is most often found in human cancers among the ~ 70 F-box genes identified in the human genome [34], meaning that approximately 6% of all cancers contain FBXW7 mutations [35] and supporting the role of FBXW7 as a tumor suppressor. To date, several mechanisms have been shown to regulate FBXW7 gene expression, including non-coding microRNA [36], DNA modification [37], histone modification [38], dimerization, and auto-ubiquitination [39]. For instance, P53 correlates with FBXW7 promoter hypermethylation, and its active form can directly bind to and stimulate FBXW7 expression [8, 40]. Similarly, EZH2 (enhancer of zeste homolog 2 polycomb repressive complex 2) is a histone methyltransferase that can silence FBXW7 function by trimethylating histone H3 at Lys27 (H3K27me3) [38]. It was recently shown that increased FBXW7 expression in mouse embryonic stem cells is facilitated by METTL5-mediated 18S ribosomal RNA m^6^A modification [41]. Altogether, these studies suggest that methylation is a crucial biological alteration that controls various mechanisms that epigenetically regulate FBXW7 expression and transcription.

Unlike DNA and histone methylation, m^6^A mRNA methylation that governs FBXW7 expression and/or function in cancers is largely unknown. This study found that FBXW7 protein levels correlate positively with METTL3 protein levels in LUAD tissues. In addition, we suggest that METTL3 initiates the m6A mRNA methylation of FBXW7. Specifically, we found that METTL3-dependent m^6^A modifications in the CDS of FBXW7 mRNA promote FBXW7 translation in LUAD, and m^6^A-modified FBXW7 enhanced antiproliferative and proapoptotic effects in LUAD, both in vitro and in vivo, validating the positive regulatory effects of METTL3 on FBXW7 expression.

Previous studies have shown that m^6^A modification is involved in many biological processes in mammals, including mRNA splicing, export, localization, translation, and stability [42]. Moreover, the m^6^A methylation of several associated proteins has been closely related to tumorigenesis in a broad range of cancer types [43]. As a “writer” in the m^6^A methyltransferase complex, METTL3 has been shown to affect various biological behaviors and, in turn, mediate carcinogenesis and development. Interestingly, METTL3 appears to act as a double-edged sword (inhibition or promotion) when exerting different molecular biological behaviors that mediate carcinogenesis and development. For instance, Deng et al. [44] found that METTL3 overexpression can inhibit the proliferation, migration, and invasion of CRC cells, whereas Li et al. [45] confirmed that METTL3 depletion suppresses CRC tumorigenesis and metastasis.

Accumulating evidence has indicated that the driver genetic cells, control pathways, and signaling networks in LUAD and LUSC differ substantially [46]. Notably, TP63 suppresses apoptosis, metastasis, and the DNA damage response in cancers and was shown to be a sensitive and specific IHC marker for distinguishing and subclassifying LUAD and/or LUSC. Moreover, TP63 has been associated with a worse prognosis in LUAD and a better prognosis in LUSC [47]. Overall, the stark differences between their genomic and pathway networks suggest that LUAD and LUSC are distinct diseases with different molecular mechanisms of initiation and epigenetic regulatory modifications. Therefore, although METTL3 has been reported to exert a cancer-promoting function in NSCLC, it is crucial to elucidate the unexpected tumor-suppressive role of m^6^A modification initiated by METTL3 in LUAD. In this study, analysis of TCGA database indicated that METTL3 was downregulated in LUAD and correlated with poor prognosis, making it a prognostic factor for patients with LUAD. In addition, METTL3 expression was significantly lower in LUAD specimens than in adjacent normal tissues and was accompanied by downregulated FBXW7 expression. Therefore, our results suggest that these rare effects of METTL3 are mainly based on increased FBXW7 m^6^A modification.

Unfortunately, some previous studies’ findings are inconsistent with our conclusions; however, these results may be explained by a failure to consider a valid genetic background. Recently, Jin et al. found that METTL3 plays an oncogenic role in NSCLC by enhancing YAP translation and promoting YAP activity via miR-19143p to stimulate drug resistance and metastasis in NSCLC cells (A549 and H1299) [48]. We propose that this phenotypic difference likely reflects the target specificity of METTL3-mediated m^6^A modified RNAs in different types of lung cancer cells, as well as tremendous genetic or epigenetic heterogeneity. The HCC827 and PC9 cells used in this study were considered a representative model of LUAD and differ substantially from LUSC at the molecular, pathological, and clinical levels [49–51]. Importantly, we found drastic differences when comparing our results with previously published METTL3 target proteins in other cancer cell lines, illustrating the high target specificity of m^6^A-modified proteins [52] and rationality of the observed inconsistencies.

There were also some limitations to this study. First, we collected an insufficient number of human LUAD samples, reducing the strength of our conclusion. Second, m^6^A methylation site prediction and functional verification should be performed at multiple levels to confirm the METTL3-dependent epigenetic regulatory mechanism of FBXW7. Ultimately, these are important issues that should be addressed in our future research, as the role of METTL3 and FBXW7 in LUAD requires further clarification.

**Fig. 7:**
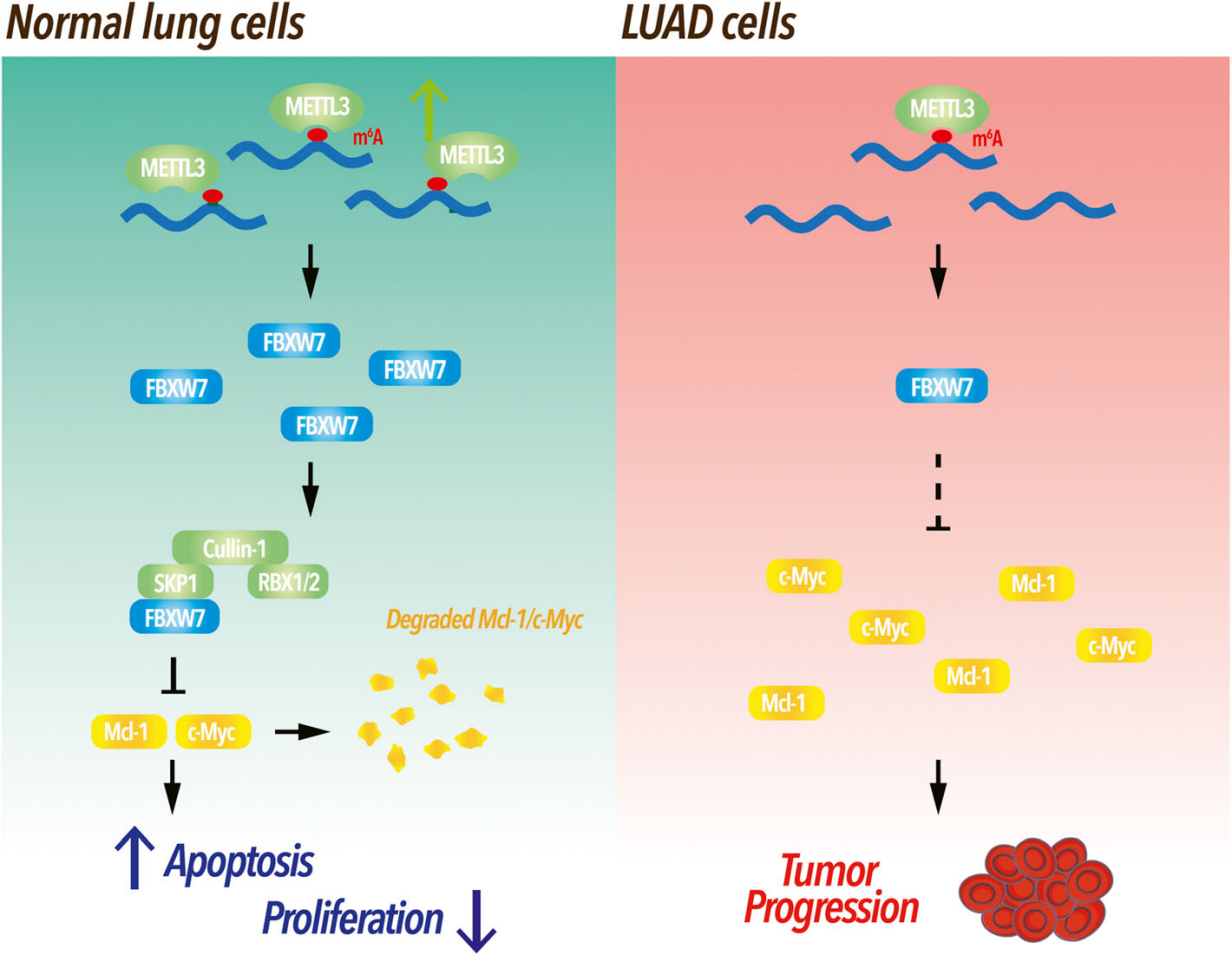
Proposed model for the function and mechanism of m^6^A-modified FBXW7 in LUAD. Mechanism of m^6^A-modified FBXW7-inhibited lung adenocarcinoma (LUAD) growth. FBXW7 may function as a tumor suppressor regulated by METTL3, which installs m^6^A modifications in the CDS of FBWX7 mRNA and inhibits cell proliferation, ultimately promoting apoptosis via FBXW7 to suppress growth in LUAD

Our study collectively reported a novel epigenetic mechanism and compelling evidence that METTL3 may enhance the translational efficiency and tumor suppressor function of FBXW7 via a multi-step process involving mRNA m^6^A methylation. Moreover, we identified abnormally m^6^A-modified FBXW7 as a vulnerability of LUAD malignancies and provided a molecular basis for m^6^A modification agonists of FBXW7 mRNA for future use in clinical research (Fig. 7).

## Conclusions

Our findings indicate that FBXW7 expression is positively associated with METTL3 at the protein level and exerted a crucial tumor suppressor function in carcinogenesis. In addition, METTL3 increased the m^6^A modification of the CDS of FBXW7 mRNA and, in turn, enhanced FBXW7 translation in LUAD. Thus, our data provide key insights into the anticancer roles of METTL3-mediated m^6^A modification and revealed its potential regulatory mechanism, suggesting targeting the METTL3-dependent m^6^A-modification of FBXW7 as a promising therapeutic strategy for the prevention and treatment of LUAD.

## Supplementary Information

**Additional file 1: Figure S1.** m^6^A methylation of FBXW7 mRNA by METTL3. **Figure S2.** Correlation between METTL3 downregulation and unfavorable prognosis in patients with lung adenocarcinoma. **Figure S3.** FBXW7 overexpression rescued the anti-tumor phenotype impaired by METTL3 knockdown in vitro. **Figure S4.** m^6^A-methylated motifs in the coding sequence promote FBXW7 translation. **Figure S5.** Prediction score of m^6^A distribution in the non-small cell lung cancer cell line according to the sequence-based RNA adenosine methylation site predictor (SRAMP) algorithm. **Figure S6.** Histogram showing the differences of each cell group in Fig. 3a.

**Additional file 2: Table S1.** qRT-PCR primer sequences. **Table S2.** Knockdown sequences. **Table S3.** Primary antibodies used in this study. **Table S4.** The SRAMP algorithm was performed on the FBXW7 sequence of non-small cell lung cancer cell lines to predict m^6^A sites.

## Data Availability

All data generated or analyzed during the study are included in this published article.

## Abbreviations

METTL3: Methyltransferase-like 3
LUAD: Lung adenocarcinoma
IHC: Immunohistochemical
m^6^A: N6-methyladenosine
MeRIP: m^6^A RNA immunoprecipitation
FBXW7: F-Box and WD repeat domain-containing 7
qRT-PCR: Quantitative real-time quantitative polymerase chain reaction
RIP: RNA Immunoprecipitation
CDS: Coding sequence
UTR: Untranslated region

## References

[1] Bray F, Ferlay J, Soerjomataram I, Siegel R, Torre L, Jemal A (2018). Global cancer statistics 2018: GLOBOCAN estimates of incidence and mortality worldwide for 36 cancers in 185 countries. CA Cancer J Clin.

[2] Lee J, Park S, Park H, Kim S, Lee J, Lee J (2019). Tracing Oncogene Rearrangements in the Mutational History of Lung Adenocarcinoma. Cell.

[3] Zheng N, Zhou Q, Wang Z, Wei W (2016). Recent advances in SCF ubiquitin ligase complex: clinical implications. Biochim Biophys Acta.

[4] Davis RJ (2014). Tumor suppression by the Fbw7 ubiquitin ligase: mechanisms and opportunities. Cancer Cell.

[5] Yumimoto K, Nakayama K. Recent insight into the role of FBXW7 as a tumor suppressor. Semin Cancer Biol. 2020;67:1–15. 10.1016/j.semcancer.2020.02.017 32113998

[6] Wang J, Wang H, Peters M, Ding N, Ribback S, Utpatel K (2019). Loss of Fbxw7 synergizes with activated Akt signaling to promote c-Myc dependent cholangiocarcinogenesis. J Hepatol.

[7] Gstalder C, Liu D, Miao D, Lutterbach B, DeVine A, Lin C, et al. Fbxw7Inactivation of Impairs dsRNA Sensing and Confers Resistance to PD-1 Blockade. Cancer discovery. 2020;10(9):1296–311. 10.1158/2159-8290.CD-19-1416 PMC8802534 32371478

[8] Yokobori T, Mimori K, Iwatsuki M, Ishii H, Onoyama I, Fukagawa T (2009). p53-altered FBXW7 expression determines poor prognosis in gastric cancer cases. Cancer Res.

[9] Lu K, Zhou X (2007). The prolyl isomerase PIN1: a pivotal new twist in phosphorylation signalling and disease. Nat Rev Mol Cell Biol.

[10] Balamurugan K, Sterneck E (2013). The many faces of C/EBPδ and their relevance for inflammation and cancer. Int J Biol Sci.

[11] Mansour M, Sanda T, Lawton L, Li X, Kreslavsky T, Novina C (2013). The TAL1 complex targets the FBXW7 tumor suppressor by activating miR-223 in human T cell acute lymphoblastic leukemia. J Exp Med.

[12] Peer E, Rechavi G, Dominissini D (2017). Epitranscriptomics: regulation of mRNA metabolism through modifications. Curr Opin Chem Biol.

[13] Perry RP, Kelley DE (1974). Existence of methylated messenger RNA in mouse L cells. Cell..

[14] Bokar JA, Shambaugh ME, Polayes D, Matera AG, Rottman FM. Purification and cDNA cloning of the AdoMet-binding subunit of the human mRNA (N6-adenosine)-methyltransferase. Rna-a Publication of the Rna Society. 1997;3(11):1233–47. PMC1369564 9409616

[15] Wang X (2015). N (6)-methyladenosine modulates messenger RNA translation efficiency. Cell..

[16] Wang X (2014). N6-methyladenosine-dependent regulation of messenger RNA stability. Nature..

[17] Jia G, Fu Y, Zhao X, Dai Q, Zheng G, Yang Y (2011). N6-methyladenosine in nuclear RNA is a major substrate of the obesity-associated FTO. Nat Chem Biol.

[18] Zheng G, Dahl J, Niu Y, Fedorcsak P, Huang C, Li C (2013). ALKBH5 is a mammalian RNA demethylase that impacts RNA metabolism and mouse fertility. Mol Cell.

[19] Meyer K, Patil D, Zhou J, Zinoviev A, Skabkin M, Elemento O (2015). 5′ UTR m (6) A promotes cap-independent translation. Cell..

[20] Huang H, Weng H, Sun W, Qin X, Shi H, Wu H (2018). Recognition of RNA N-methyladenosine by IGF2BP proteins enhances mRNA stability and translation. Nat Cell Biol.

[21] Li Z, Weng H, Su R, Weng X, Zuo Z, Li C (2017). FTO plays an oncogenic role in acute myeloid leukemia as a N-Methyladenosine RNA Demethylase. Cancer Cell.

[22] Zhang S (2017). mA Demethylase ALKBH5 Maintains Tumorigenicity of Glioblastoma Stem-like Cells by Sustaining FOXM1 Expression and Cell Proliferation Program. Cancer cell.

[23] Cai X, Wang X, Cao C, Gao Y, Zhang S, Yang Z (2018). HBXIP-elevated methyltransferase METTL3 promotes the progression of breast cancer via inhibiting tumor suppressor let-7g. Cancer Lett.

[24] Chen M, Wei L, Law CT, Tsang FH (2018). RNA N6-methyladenosine methyltransferase-like 3 promotes liver cancer progression through YTHDF2-dependent posttranscriptional silencing of SOCS2. Hepatology (Baltimore, Md).

[25] Liu J, Eckert MA, Harada BT, Liu SM (2018). mA mRNA methylation regulates AKT activity to promote the proliferation and tumorigenicity of endometrial cancer. Nat Cell Biol.

[26] Wang T, Kong S, Tao M, Ju S (2020). The potential role of RNA N6-methyladenosine in Cancer progression. Mol Cancer.

[27] Wang S, Chai P, Jia R, Jia R (2018). Novel insights on mA RNA methylation in tumorigenesis: a double-edged sword. Mol Cancer.

[28] Ye M, Zhang Y, Zhang X, Zhang J, Jing P, Cao L (2017). Targeting FBW7 as a strategy to overcome resistance to targeted therapy in non-small cell lung Cancer. Cancer Res.

[29] Zhang Y, Zhang X, Ye M, Jing P, Xiong J, Han Z (2018). FBW7 loss promotes epithelial-to-mesenchymal transition in non-small cell lung cancer through the stabilization of snail protein. Cancer Lett.

[30] Cennamo A, Falsetto A, De Pascale V, Gallo G, Della Corte M (2001). A rare gastric ulcer complication: the gastrocolic fistula. A case report. Chir Ital.

[31] Liu J, Lichtenberg T, Hoadley K, Poisson L, Lazar A, Cherniack A (2018). An Integrated TCGA Pan-Cancer Clinical Data Resource to Drive High-Quality Survival Outcome Analytics. Cell.

[32] Zhou Y, Zeng P, Li Y, Zhang Z, Cui Q (2016). SRAMP: prediction of mammalian N6-methyladenosine (m6A) sites based on sequence-derived features. Nucleic Acids Res.

[33] Tang Z, Kang B, Li C, Chen T, Zhang Z (2019). GEPIA2: an enhanced web server for large-scale expression profiling and interactive analysis. Nucleic Acids Res.

[34] Jin J, Cardozo T, Lovering RC, Elledge SJ, Pagano M, Harper JW (2004). Systematic analysis and nomenclature of mammalian F-box proteins. Genes Dev.

[35] Forbes SA, Beare D, Boutselakis H, Bamford S, Bindal N, Tate J (2017). COSMIC: somatic cancer genetics at high-resolution. Nucleic Acids Res.

[36] Wang Y, Liu Z, Yao B (2017). Long non-coding RNA CASC2 suppresses epithelial-mesenchymal transition of hepatocellular carcinoma cells through CASC2/miR-367/FBXW7 axis. Molecular cancer.

[37] Akhoondi S, Lindstrom L, Widschwendter M (2010). Inactivation of FBXW7/hCDC4-β expression by promoter hypermethylation is associated with favorable prognosis in primary breast cancer. Breast Cancer Res.

[38] Zhao E, Maj T, Kryczek I, Li W, Wu K, Zhao L (2016). Cancer mediates effector T cell dysfunction by targeting microRNAs and EZH2 via glycolysis restriction. Nat Immunol.

[39] Welcker M, Larimore EA, Swanger J (2013). Fbw7 dimerization determines the specificity and robustness of substrate degradation. Genes Dev.

[40] Mao J, Perez-Losada J, Wu D, Delrosario R, Tsunematsu R, Nakayama K (2004). Fbxw7/Cdc4 is a p53-dependent, haploinsufficient tumour suppressor gene. Nature..

[41] Xing M, Liu Q, Mao C, Zeng H, Zhang X, Zhao S, et al. The 18S rRNA m A methyltransferase METTL5 promotes mouse embryonic stem cell differentiation. EMBO reports. 2020;21(10):e49863. 10.15252/embr.201949863 PMC7534618 32783360

[42] Huang H, Weng H, Chen J (2020). mA modification in coding and non-coding RNAs: roles and therapeutic implications in Cancer. Cancer Cell.

[43] Zhou Z, Lv J, Yu H, Han J, Yang X, Feng D (2020). Mechanism of RNA modification N6-methyladenosine in human cancer. Mol Cancer.

[44] Deng R, Cheng Y, Ye S, Zhang J, Huang R, Li P (2019). mA methyltransferase METTL3 suppresses colorectal cancer proliferation and migration through p38/ERK pathways. OncoTargets and therapy.

[45] Li T, Hu PS, Zuo Z, Lin JF (2019). METTL3 facilitates tumor progression via an mA-IGF2BP2-dependent mechanism in colorectal carcinoma. Molecular cancer.

[46] Relli V, Trerotola M, Guerra E, Alberti S (2019). Abandoning the notion of non-small cell lung Cancer. Trends Mol Med.

[47] Gurda G, Zhang L, Wang Y, Chen L, Geddes S, Cho W (2015). Utility of five commonly used immunohistochemical markers TTF-1, Napsin A, CK7, CK5/6 and P63 in primary and metastatic adenocarcinoma and squamous cell carcinoma of the lung: a retrospective study of 246 fine needle aspiration cases. Clin Transl Med.

[48] Jin D, Guo J, Wu Y, Du J, Yang L, Wang X (2019). mA mRNA methylation initiated by METTL3 directly promotes YAP translation and increases YAP activity by regulating the MALAT1-miR-1914-3p-YAP axis to induce NSCLC drug resistance and metastasis. J Hematol Oncol.

[49] Liu J, Yang X, Shi W (2014). Identifying differentially expressed genes and pathways in two types of non-small cell lung cancer: adenocarcinoma and squamous cell carcinoma. Genet Mol Res.

[50] Chang Y, Chen C, Chen H, Yang P (2015). Pathway-based gene signatures predicting clinical outcome of lung adenocarcinoma. Sci Rep.

[51] Campbell J, Yau C, Bowlby R, Liu Y, Brennan K, Fan H (2018). Genomic, Pathway Network, and Immunologic Features Distinguishing Squamous Carcinomas. Cell reports.

[52] Zeng C, Huang W, Li Y, Weng H (2020). Roles of METTL3 in cancer: mechanisms and therapeutic targeting. J Hematol Oncol.

