# Supplementary material for: METTL3-mediated m6A mRNA modification of FBXW7 suppresses lung adenocarcinoma
Source: J Exp Clin Cancer Res. 2021 Mar 6;40:90. doi: 10.1186/s13046-021-01880-3 (PMC7936500; doi:10.1186/s13046-021-01880-3)
Supplement: Supplementary file 1 — Additional file 1: Figure S1. m6A methylation of FBXW7 mRNA by METTL3. Figure S2. Correlation between METTL3 downregulation and unfavorable prognosis in patients with lung adenocarcinoma. Figure S3. FBXW7 overexpression rescued the anti-tumor phenotype impaired by METTL3 knockdown in vitro. Figure S4. m6A-methylated motifs in the coding sequence promote FBXW7 translation. Figure S5. Prediction score of m6A distribution in the non-small cell lung cancer cell line according to the sequence-based RNA adenosine methylation site predictor (SRAMP) algorithm. Figure S6. Histogram showing the differences of each cell group in Fig. 3a. [file 13046_2021_1880_MOESM1_ESM.docx]

**Additional file 1**

**
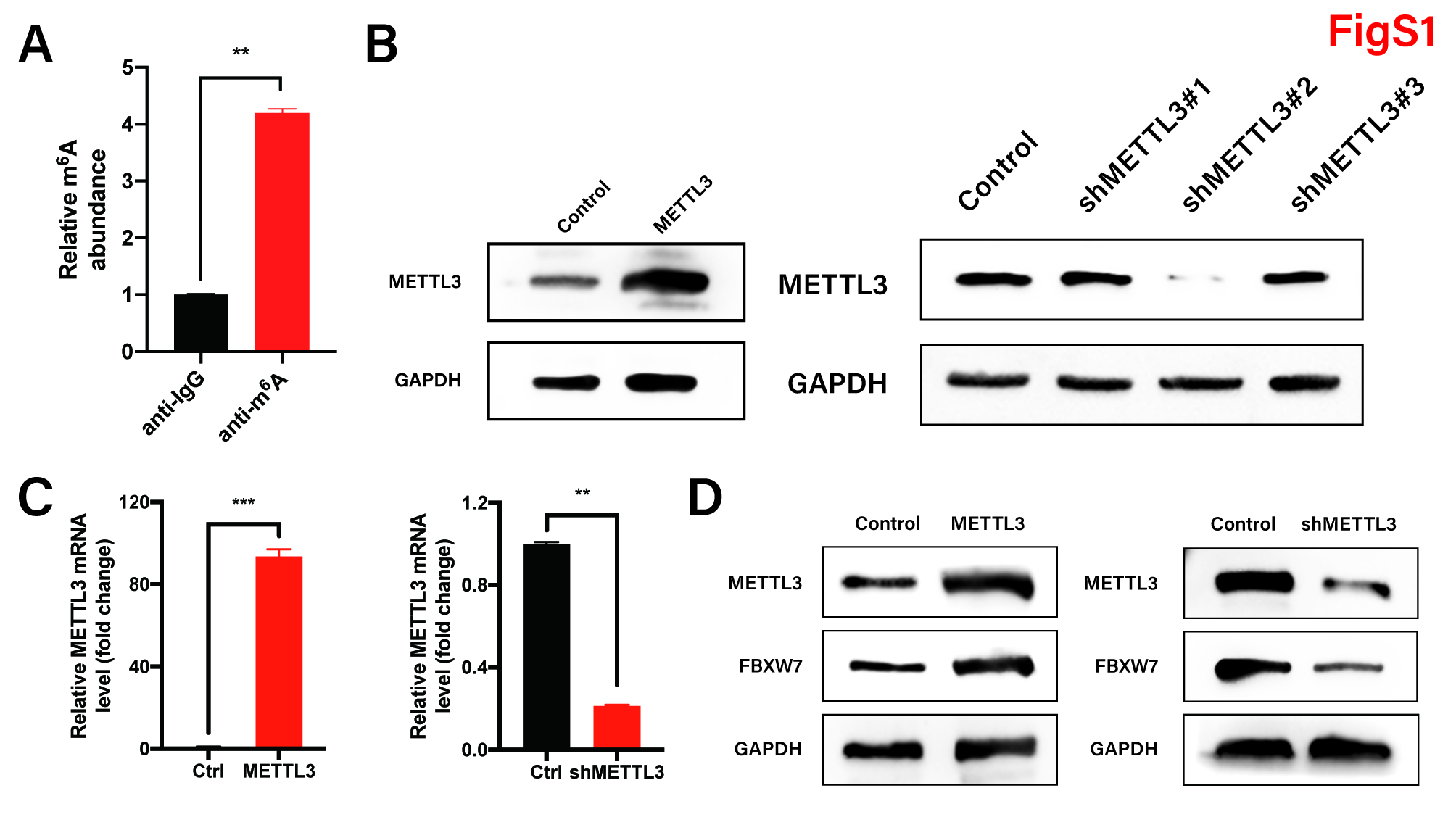
**

Figure S1. m^6^A methylation of FBXW7 mRNA by METTL3. (**A**) m^6^A abundance on FBXW7 mRNA in PC9 cells detected by me-RIP. (**B**) METTL3 overexpression or knockdown effect was verified by western blotting in HCC827 cells. (**C**) METTL3 overexpression or knockdown effect was verified by quantitative real-time PCR in HCC827 cells. (**D**) Western blotting of FBXW7 and METTL3 in METTL3 overexpression or knockdown PC9 cells. (**E**) Representative IHC staining showing METTL3 and FBXW7 in LUAD and adjacent normal tissue. Bars = means ± SD. ***p* < 0.01

**
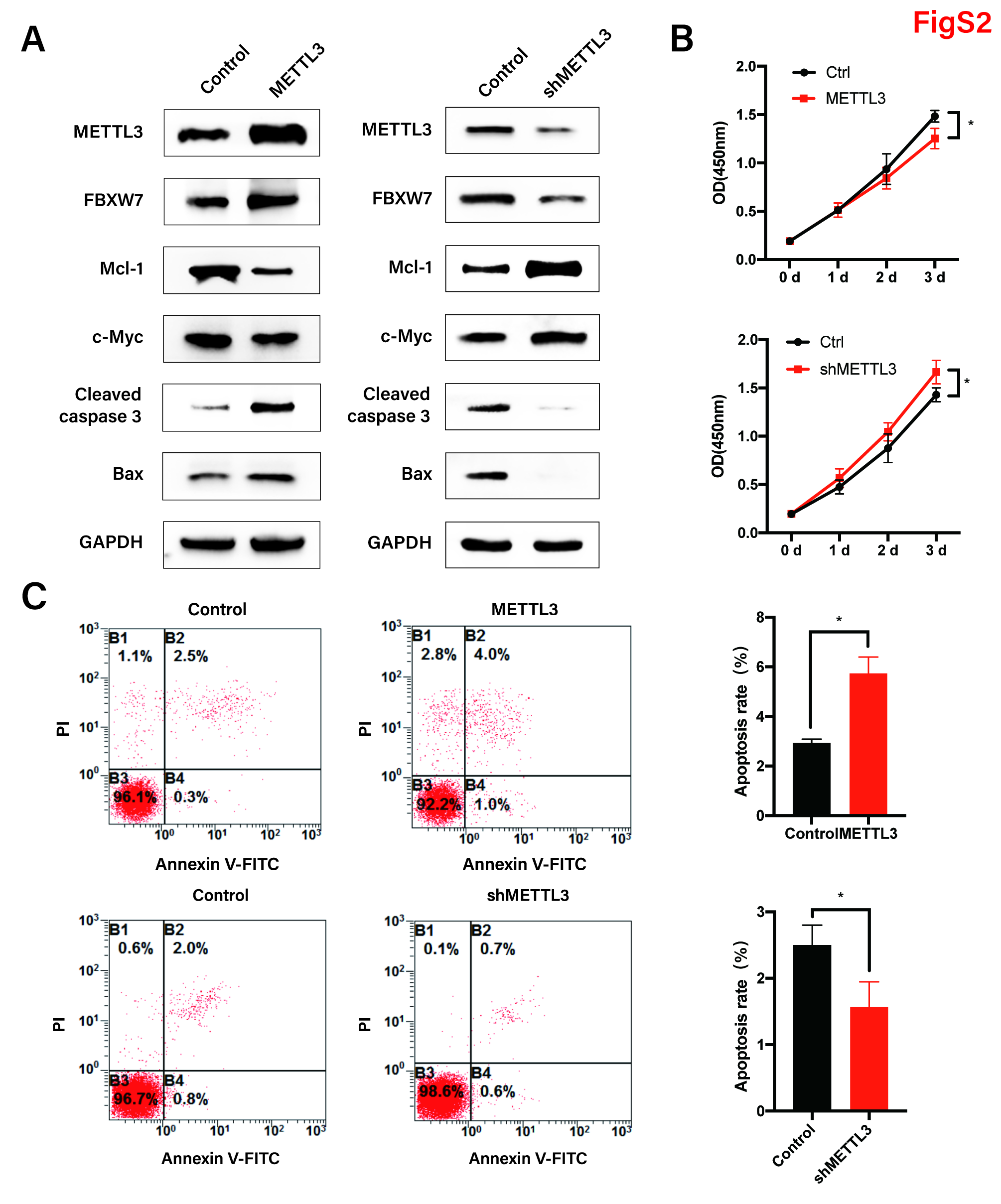
**

Figure S2. Correlation between METTL3 downregulation and unfavorable prognosis in patients with LUAD. (**A**) Western blotting of Mcl-1, c-Myc, Cleaved caspase 3, Bax, METTL3 and FBXW7 protein expression in METTL3 overexpression or depletion PC9 cells. (**B，C**) Overexpression or depletion of METTL3 affected the apoptosis and proliferation of PC9 cells. Bars = means ± SD. **p* < 0.05

**
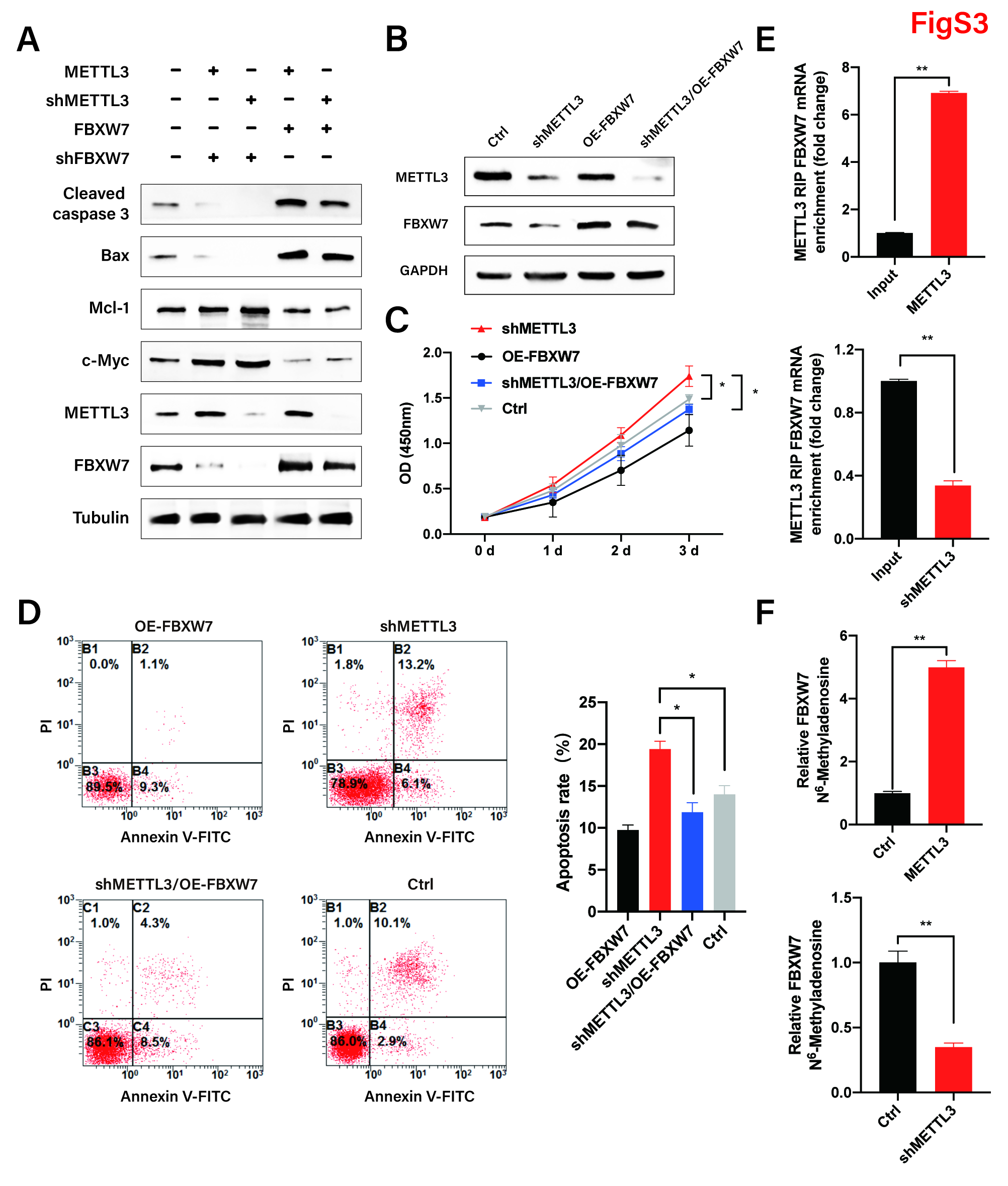
**

Figure S3. FBXW7 overexpression rescued the anti-tumor phenotype impaired by METTL3 knockdown *in vitro*. (**A**) The relative protein levels of Mcl-1, c-Myc, Cleaved caspase 3, Bax, METTL3 and FBXW7 was detected in PC9 cells with indicated plasmids. **(B)** The expressions of METTL3 and FBXW7 were analyzed by Western blot in PC9 cells with indicated plasmids. **(C, D)** Effects of overexpressing FBXW7 in shMETTL3 PC9 cells. FBXW7 partially restored the cell apoptosis and inhibition of cell proliferation in PC9 cells, which were reduced by METTL3 knockdown. **(E)** RNA immunoprecipitation (RIP) assay demonstrated Association of METTL3 and FBXW7 mRNA in PC9 cells. (**F**) The m^6^A modification levels of FBXW7 in PC9 cells was determined by using MeRIP-qPCR. Bars = means ± SD. **p* < 0.05; and ***p* < 0.01


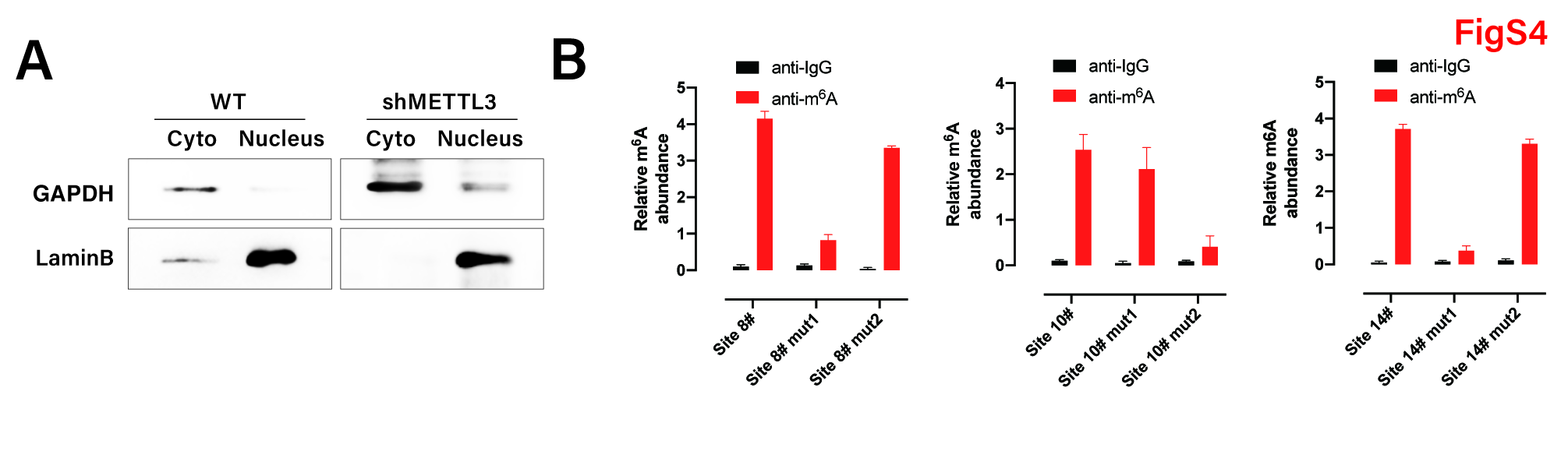


Figure S4. m^6^A-methylated motifs in the CDS promote FBXW7 translation. (**A**) The cytoplasmic and nuclear fractions of control or shMETTL3 HCC827 cells were separated and GAPDH and LaminB were used as internal references for the cytoplasmic and nuclear fractions, respectively. (**B**) The m^6^A level of predictive m^6^A sites were analyzed by MeRIP-qPCR. Samples are normalized to non-crosslinked input to remove background noise. Anti-IgG antibody is used as control. Bars = means ± SD.

**
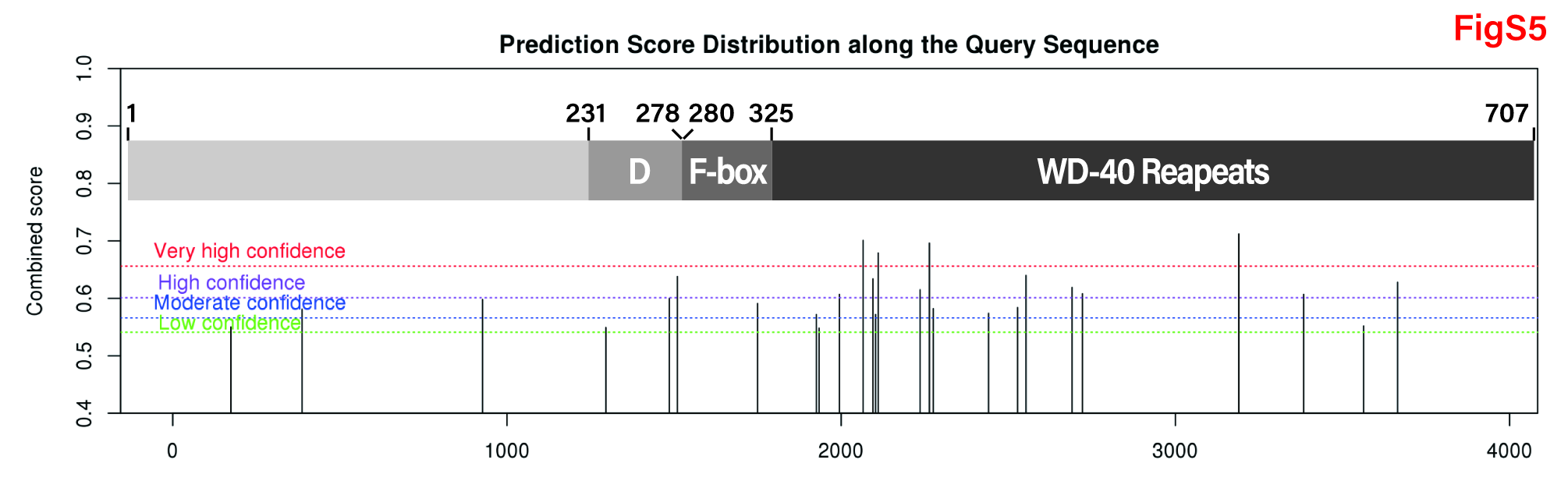
**

Figure S5. Prediction score of m^6^A distribution along NSCLC cell line according to the sequence-based RNA adenosine methylation site predictor (SRAMP) algorithm. Y axis presents the combined score at different levels of high (H), moderate (M), and low (L) probability. Vertical bars show the score for predictive m^6^A sites. X axis shows FBXW7 genome in base pair resolution. Annotated features of FXBW7 genome are shown above the X axis.


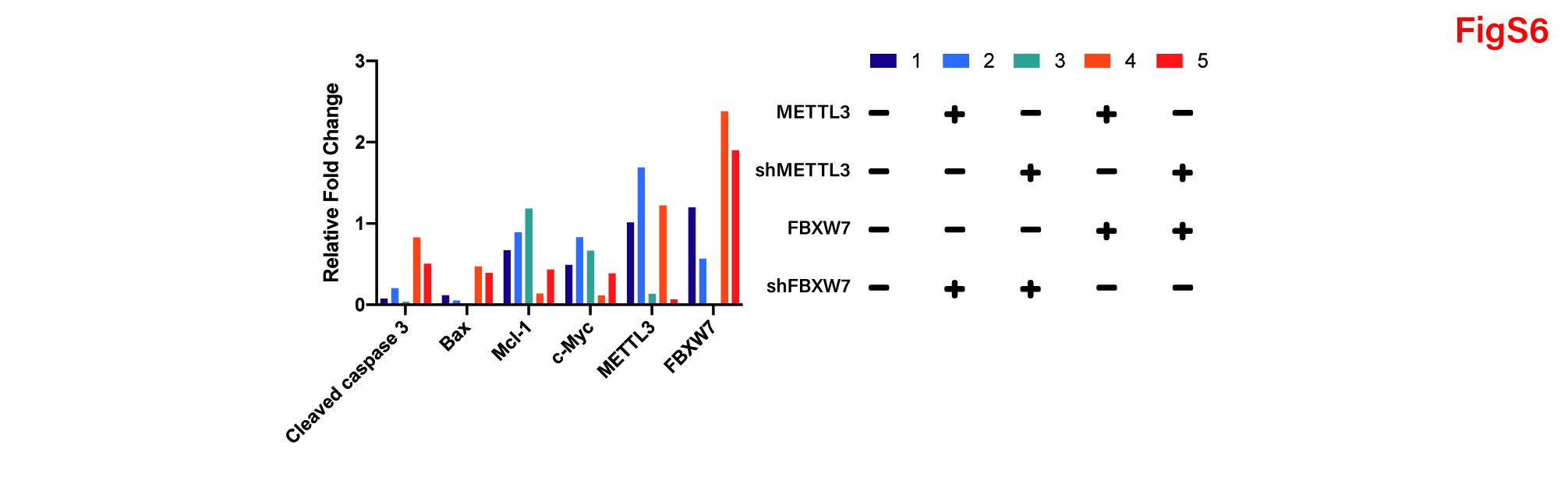


Figure S6. Histogram showing the differences of each cell group in Fig 3A.
