# Supplementary material for: METTL3-mediated m6A mRNA modification of FBXW7 suppresses lung adenocarcinoma
Source: J Exp Clin Cancer Res. 2021 Mar 6;40:90. doi: 10.1186/s13046-021-01880-3 (PMC7936500; doi:10.1186/s13046-021-01880-3)
Supplement: Supplementary file 2 — Additional file 2: Table S1. qRT-PCR primer sequences. Table S2. Knockdown sequences. Table S3. Primary antibodies used in this study. Table S4. The SRAMP algorithm was performed on the FBXW7 sequence of non-small cell lung cancer cell lines to predict m6A sites. [file 13046_2021_1880_MOESM2_ESM.docx]

**Additional file 2**

Supplementary Table S1. Sequences of Primer for Real-time Polymerase Chain Reaction.

| **METTL3** |  |
| --- | --- |
| Forward | 5’-AGCCTTCTGAACCA ACAGTCC-3’ |
| Reverse | 5’-CCGACCTCGAGAGCGAAAT-3’ |
| **FBXW7** |  |
| Forward | 5’-GGCCAAAATGATTCCCAGCAA-3’ |
| Reverse | 5’-ACTGGAGTTCGTGACACTGTTA-3’ |
| **FBXW7 5’UTR** |  |
| Forward | 5’-GCACTTCACCAAGCCTCAGAC-3’ |
| Reverse | 5’-CTGAGAACCCAAGGCCTGCT-3’ |
| **FBXW7 CDS** |  |
| Forward | 5’-CACGTTGCAGGGGCATACTA-3’ |
| Reverse | 5’-CTCCACATCCCAAACACGGA-3’ |
| **FBXW7 3’UTR** |  |
| Forward | 5’-TTGCCACTGAAACTTGAGCC-3’ |
| Reverse  **FBXW7 m6A Site 1#**  Forward  Reverse  **FBXW7 m6A Site 2#**  Forward  Reverse  **FBXW7 m6A Site 3#**  Forward  Reverse  **FBXW7 m6A Site 4#**  Forward  Reverse  **FBXW7 m6A Site 5#**  Forward  Reverse  **FBXW7 m6A Site 6#**  Forward  Reverse  **FBXW7 m6A Site 7#**  Forward  Reverse  **FBXW7 m6A Site 8#**  Forward  Reverse  **FBXW7 m6A Site 9#**  Forward  Reverse  **FBXW7 m6A Site 10#**  Forward  Reverse  **FBXW7 m6A Site 11#**  Forward  Reverse  **FBXW7 m6A Site 12#**  Forward  Reverse  **FBXW7 m6A Site 13#**  Forward  Reverse  **FBXW7 m6A Site 14#**  Forward  Reverse  **FBXW7 m6A Site 15#**  Forward  Reverse | 5’-TCTCCACAGAACAGGCAAGT-3’  5’-CGCCGCGGCTCTTTTCTAAA-3’  5’-TGCCCACAGAGAGCAGTTCC-3’  5’-TCTGTCCAGCCACCTACAGG-3’  5’-ACTGGGGTTCTATCACTTGCATCA-3’  5’-TCTGTCCAGCCACCTACAGG-3’  5’-ACTGGGGTTCTATCACTTGCATCA-3’  5’-CTGGAGGCGAGGAGAACTCAAATC-3’  5’-TGCCTGTGACTGCTGACCAAAC-3’  5’-AGTGGGACATACAGGTGGAGT-3’  5’-ACGCACAGTGGAAGTATGCC-3’  5’-CTACACACGTTGCAGGGGCATAC-3’  5’-CCTGTCTCCACATCCCAAACACG-3’  5’-CTTGTCTCTGGGAATGCAGATTCT-3’  5’-TGCTTGTTGGGACCTTGCAA-3’  5’-TTGCAAGGTCCCAACAAGCA-3’  5’-CCATCATCTGAGCTGGTAATTACAAAG-3’  5’-ACGGGTGAATTTATTCGAAACCTAGTC-3’  5’-GCTTTGTGTTTGAGGCTCTGATCC-3’  5’-GAGTTGTGTGGCGGATCAGAGC-3’  5’-CCAGCAGCTTGGTTTCTTCAGTCC-3’  5’-TGCTGGTGCTGGACTTTGATGTG-3’  5’-GGGGAAGGGCAGGGAGTATATCG-3’  5’-GAGGCGTCTGCTGTCTCATCAC-3’  5’-CTGCCCAATGACCACTGGAGAAG-3’  5’-ACCACTGACAGCTAGACACCT-3’  5’-TGACTTTTTGTTGCAGATGCTCTCT-3’  5’-TGCCACTGAAACTTGAGCCA-3’  5’-ACTTGCAGGCAAACTTCACTCT-3’  5’-AGTAAAGGCGTCACATGCAAACA-3’  5’-ACAGTTCAGCTTGAGTTGTGGC-3’ |
| **GAPDH** |  |
| Forward | 5’-CTCCTCCTGTTCGA CAGTCAGC-3’ |
| Reverse | 5’-CCCAATACGACCAA ATCCGTT-3’ |

Supplementary Table S2. Sequences of knockdown.

| **shFBXW7** | 5’-AGAGAAATTGCTTGCTTTA-3ʹ |
| --- | --- |

Supplementary Table S3. List of Primary Antibodies Used In the study.

| Anti-METTL3  antibody | Rabbit monoclonal | abcam, # ab195352 | WB: 1:1000  IHC-P: 1:500 |
| --- | --- | --- | --- |
| Anti-FBXW7  antibody | Rabbit polyclonal | Bethyl, A301-720A | WB: 1:1000  IHC-P: 1:1000 |
| Anti-FBXW7  antibody | Mouse monoclonal | sc-293423 | IHC-P: 1:500 |
| Anti-WTAP  antibody | Rabbit monoclonal | abcam, #ab195380 | WB: 1:1000 |
| Anti-Lamin B1 antibody | Rabbit monoclonal | abcam, #ab16048 | WB: 1:1000 |
| Anti-Tubullin antibody | Rabbit monoclonal | abcam, #ab7291 | WB: 1:1000 |
| Anti-Bax  Antibody | Rabbit monoclonal | CST, #14796 | WB: 1:1000 |
| Anti-Cleaved Caspase-3  Antibody | Rabbit monoclonal | CST, #9661 | WB: 1:1000 |
| Anti-c-Myc  Antibody | Rabbit monoclonal | CST, #18583 | WB: 1:1000 |
| Anti-Mcl-1  Antibody | Rabbit monoclonal | CST, #94296 | WB: 1:1000 |
| Anti-Bcl-2  Antibody | Rabbit monoclonal | CST, #15071 | WB: 1:100 |
| Anti-IgG  antibody | Rabbit monoclonal | beyotime, #A7016 | RIP: 1:100 |
| Anti-m6A  antibody | Mouse monoclonal | Abcam, #ab208577 | MeRIP: 1:150 |
| Anti-GAPDH Antibody | Rabbit monoclonal | CST, #5174 | WB: 1:1000 |

Supplementary Table S4. Output of SRAMP algorithm performed on FBXW7 sequence of NSCLC cell lines to predict m6A sites.

| Site# | Site | Position | Sequence context | Score  (binary) | Score  (knn) | Score  (spectrum) | Score  (combined) | Probability |
| --- | --- | --- | --- | --- | --- | --- | --- | --- |
| 1 | 1 | 174 | GGCUUUUGGAAAUGAAUCAGGAACUGCUCUCUGUGGGCAGCAAAA | 0.574 | 0.493 | 0.523 | 0.55 | Low |
| 2 | 2 | 387 | GCCAAAAUGAUUCCCAGCAAGGACAGUUGGAAGAAAACAAUAAUA | 0.709 | 0.748 | 0.384 | 0.581 | Moderate |
| 3 | 3 | 927 | AGAAAUUGCUUGCUUUAGAUGAACUCAUUGAUAGUUGUGAACCAA | 0.636 | 0.725 | 0.529 | 0.598 | Moderate |
| 4 | 4 | 1296 | AAUCUCCUAAGGUGCUGAAAGGACAUGAUGAUCAUGUGAUCACAU | 0.602 | 0.457 | 0.489 | 0.549 | Low |
| 5 | 5 | 1486 | CAUUAGUGGAUCUACAGAUCGGACACUCAAAGUGUGGAAUGCAGA | 0.58 | 0.699 | 0.614 | 0.6 | Moderate |
|  | 6 | 1510 | ACUCAAAGUGUGGAAUGCAGAGACUGGAGAAUGUAUACACACCUU | 0.682 | 0.676 | 0.574 | 0.638 | High |
| 6 | 7 | 1750 | GGUAAAGGUGUGGGAUCCAGAGACUGAAACCUGUCUACACACGUU | 0.692 | 0.603 | 0.45 | 0.591 | Moderate |
|  | 8 | 1926 | AGUCGUUAACAAGUGGAAUGGAACUCAAAGACAAUAUUCUUGUCU | 0.565 | 0.599 | 0.579 | 0.572 | Moderate |
| 7 | 9 | 1934 | ACAAGUGGAAUGGAACUCAAAGACAAUAUUCUUGUCUCUGGGAAU | 0.551 | 0.415 | 0.561 | 0.548 | Low |
|  | 10 | 1995 | AAAUCUGGGAUAUCAAAACAGGACAGUGUUUACAAACAUUGCAAG | 0.663 | 0.76 | 0.512 | 0.607 | High |
| 8 | 11 | 2066 | ACCUGUUUACAGUUCAACAAGAACUUUGUAAUUACCAGCUCAGAU | 0.748 | 0.808 | 0.624 | 0.701 | Very high |
|  | 12 | 2095 | AAUUACCAGCUCAGAUGAUGGAACUGUAAAACUAUGGGACUUGAA | 0.654 | 0.526 | 0.619 | 0.634 | High |
| 9 | 13 | 2103 | GCUCAGAUGAUGGAACUGUAAAACUAUGGGACUUGAAAACGGGUG | 0.552 | 0.368 | 0.624 | 0.572 | Moderate |
|  | 14 | 2111 | GAUGGAACUGUAAAACUAUGGGACUUGAAAACGGGUGAAUUUAUU | 0.721 | 0.679 | 0.622 | 0.679 | Very high |
| 10 | 15 | 2236 | UGCAGUUGGGAGUCGGAAUGGGACUGAAGAAACCAAGCUGCUGGU | 0.703 | 0.62 | 0.493 | 0.615 | High |
|  | 16 | 2264 | GAAACCAAGCUGCUGGUGCUGGACUUUGAUGUGGACAUGAAGUGA | 0.797 | 0.845 | 0.539 | 0.696 | Very high |
| 11 | 17 | 2276 | CUGGUGCUGGACUUUGAUGUGGACAUGAAGUGAAGAGCAGAAAAG | 0.598 | 0.624 | 0.555 | 0.582 | Moderate |
|  | 18 | 2441 | AACAGAUUGAAAAGACCUACAGACUAAGAAGGAAAAGAAGAAGAG | 0.659 | 0.551 | 0.459 | 0.574 | Moderate |
| 12 | 19 | 2528 | CACAUAAAAGGCUUCACUUUUGACUGAGGGCAGCUUUGCAAAAUG | 0.527 | 0.791 | 0.638 | 0.584 | Moderate |
|  | 20 | 2553 | GAGGGCAGCUUUGCAAAAUGAGACUUUCUAAAUCAAACCAGGUGC | 0.773 | 0.56 | 0.468 | 0.64 | High |
| 13 | 21 | 2691 | ACAGCUAGACACCUAGAAAGGAACUGCAAUAAUAUCAAAACAAGU | 0.636 | 0.535 | 0.606 | 0.619 | High |
|  | 22 | 2722 | AUAUCAAAACAAGUACUGGUUGACUUUCUAAUUAGAGAGCAUCUG | 0.577 | 0.682 | 0.643 | 0.608 | High |
| 14 | 23 | 3190 | CUAGUUUGGUUAUGGAAAAAAGACUUUUUGCCACUGAAACUUGAG | 0.779 | 0.63 | 0.632 | 0.712 | Very high |
|  | 24 | 3384 | UACUUGCCUGUUCUGUGGAGAAACUUUUCUUUUUGAGGGCUGUGG | 0.588 | 0.274 | 0.675 | 0.607 | High |
| 15 | 25 | 3563 | CUGAGUAACUGACAAAGCAGAAACUAUUCAGUUUUUGUAGUAAAG | 0.567 | 0.457 | 0.545 | 0.552 | Low |
|  | 26 | 3665 | CAAGAGCCACAACUCAAGCUGAACUGUGAAAGUGGUUUAACACUG | 0.715 | 0.754 | 0.493 | 0.628 | High |
